# Fluoro Nitrenoid Complexes FN=MF_2_ (M=Co, Rh, Ir): Electronic Structure Dichotomy and Formation of Nitrido Fluorides N≡MF_3_

**DOI:** 10.1002/anie.202010950

**Published:** 2020-10-15

**Authors:** Tony Stüker, Thomas Hohmann, Helmut Beckers, Sebastian Riedel

**Affiliations:** ^1^ Anorganische Chemie Institut für Chemie und Biochemie Freie Universität Berlin 14195 Berlin Germany

**Keywords:** ab-initio calculations, high oxidation states, matrix isolation, N ligands, transition metals

## Abstract

The fluoronitrenoid metal complexes FNCoF_2_ and FNRhF_2_ as well as the first ternary Rh^VI^ and Ir^VI^ complexes NIrF_3_ and NRhF_3_ are described. They were obtained by the reaction of excited Group‐9 metal atoms with NF_3_ and their IR spectra, isolated in solid rare gases (neon and argon), were recorded. Aided by the observed ^14/15^N isotope shifts and quantum‐chemical predictions, all four stretching fundamentals of the novel complexes were safely assigned. The F−N stretching frequencies of the fluoronitrenoid complexes FNCoF_2_ (1056.8 cm^−1^) and FNRhF_2_ (872.6 cm^−1^) are very different and their N−M bonds vary greatly. In FNCoF_2_, the FN ligand is singly bonded to Co and bears considerable iminyl/nitrene radical character, while the N−Rh bond in FNRhF_2_ is a strong double bond with comparatively strong σ‐ and π‐bonds. The anticipated rearrangement of FNCoF_2_ to the nitrido Co^VI^ complex is predicted to be endothermic and was not observed.

## Graphical abstract

The fluoronitrenoid metal complexes FNCoF_2_ and FNRhF_2_ as well as the first ternary Rh^VI^ and Ir^VI^ complexes NIrF_3_ and NRhF_3_ are reported. They were obtained by the reaction of excited Group‐9 metal atoms with NF_3_ and their IR spectra, isolated in solid rare gases, were recorded.

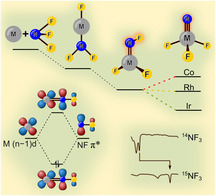

## Introduction

**Scheme 1:**
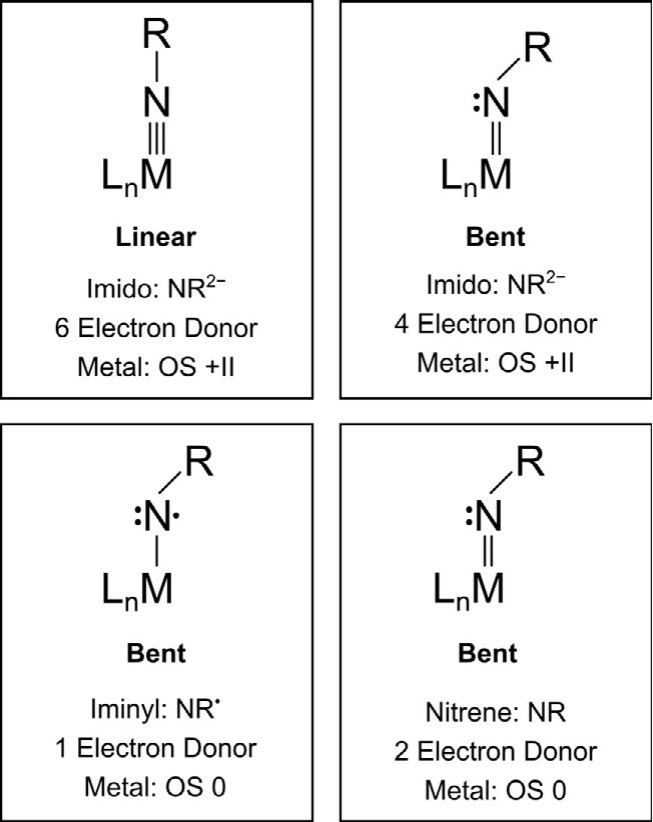
Simplified scheme^[1]^ for the interactions of a metal center and an anionic imido (formal NR^2−^, top) and a neutral nitrene (bottom) ligand, respectively, in linear and bent nitrenoid complexes. The charge of the NR ligand in the ionic (NR^2−^) and neutral (NR) approximation and the corresponding formal metal oxidation states are indicated below for uncharged donor ligands L.

Fluoronitrenoid metal complexes are underexplored compounds as only two examples have been reported so far, FNReF_5_ and FNCuF_2_.^[2]^ This is most likely due to the fact that they bear a reactive FN function and are not readily available. In general, late transition‐metal‐nitrogen multiple bonds have attracted particular interest after having been found to enable the conversion of ubiquitous C−H and C−C bonds into valuable C−N bonds as either catalysts or intermediates.[^3^, ^4^, ^5^, ^6^] Detailed knowledge of the geometry and electronic structure of such compounds are vital to elucidate the nature and mechanism of these reactions.^[4]^ While nitrido complexes usually feature a M≡N triple bond,[^4^, ^7^] the imido ligand NR^2−^ exhibits a M=N double bond in complexes with a bent M=N−R linkage (Scheme 1).^[4]^ However, it has been mentioned that the energy required to change the M=N−R angle from bent to linearity is often small, and indeed, transition metal imido complexes bearing sterically encumbered ligands to protect the reactive metal‐nitrogen multiple bond often feature short M−N bond lengths and nearly linear angles about the M−N−R linkage (Scheme 1).[^4^, ^5^]

In this bonding Scheme the anionic NR^2−^ imido ligand is expected to bear a nucleophilic character in its reactions. The polarities of both the σ and π bonds are important factors that govern the reactivity of imido complexes.[^4^, ^5^] As one moves from early to late transition metals, the binding energy of the metal d‐electrons increases, and an imido ligand becomes less nucleophilic. This applies in particular to late transition metals in higher oxidation states. In fact, the imido ligands in late 3d transition metal complexes are often so electrophilic that these complexes can be better described as metal‐nitrene complexes (Scheme 1). Formally, an imido complex differs from a nitrene complex in the formal charge of the ligand and thus in the oxidation state of the metal (Scheme 1). An imido species suggests a dianionic imido ligand (NR^2−^), whereas a neutral nitrene ligand is usually the result of a predominantly covalent nitrogen‐metal bond. In the case of neutral, covalently bound ligands, no polarization of bonding electrons towards the ligand and no or only little charge transfer from the metal to the ligand is generally to be expected. Additionally, not long‐ago a few examples were reported where an iminyl radical (^2^NR^.^) is coordinated to a transition metal.^[8]^ Metal‐imido cores containing iminyl radical ligands are proposed as reactive intermediates in a number of metal catalyzed aziridination and amination reactions.^[5]^ These and further possible interpretations of the electronic structures of terminal metal nitrenoid complexes (e.g., imido, M(RN^2−^); iminyl, M(^2^RN^.^); nitrene, M(RN); and triplet nitrene (^3^RN^..^)^[9]^ demonstrate the diversity of the metal‐nitrogen bond in M−N−R complexes.

We became interested in fluoronitrenoid complexes of the group 9 metal difluorides, F−N=MF_2_, M=Co, Rh, Ir. These simple nitrene complexes should allow a rigorous experimental and quantum‐chemical comparison of the electronic properties of the Co^IV^ complex with those of its heavier congeners. Fluoronitrenoid‐metal complexes show more complex nitrenoid‐metal binding modes and new reactivities. The fluoronitrene ligand shares some similarities with the oxygen molecule, since the nitrogen 2p electrons involved in metal‐nitrogen bonding are accommodated in a degenerate pair of π*(F−N) orbitals. Hence any metal‐to‐ligand charge transfer in a fluoronitrene complex will increase the occupancy of these π*(F−N) orbital, rendering the N−F stretching frequency a highly sensitive probe for the polarity and the strength of the N=M bond: a weak nitrogen‐metal bond in a metal‐nitrene complex result in a strong fluorine‐nitrogen bond and vice versa. Utilizing the F−N functionality and relying upon a high metal‐fluorine bond energy we have targeted the synthesis of high‐valent nitridometal trifluorides N≡MF_3_, starting from the fluoronitrene complexes by an oxidative F−N to M−F fluorine migration, by which the formal metal oxidation state will be increased by two units. As far as we know, molecular nitridometal trifluorides, NMF_3_, are known only for the early transition metals of group IV (M=Ti, Zr, Hf^[10]^) and VI (Cr, Mo, W^[11]^). Notably, the formal metal oxidation state VI in N≡MF_3_ (M=Co, Rh, Ir) is rare, with IrO_3_, Ir(η_2_‐O_2_)O_2_, IrF_6_, Rh(η_2_‐O_2_)O_2_ and RhF_6_ as the only examples.^[12]^ Terminal nitrido complexes of very high formal oxidation states have been predicted very recently,^[13]^ however, such high‐valent group 9 metals are still unknown. They would be of particular interest for cobalt, since the highest oxidation state reported for any molecular complex of cobalt is V, for example, the well‐known [Co(1‐norbornyl)_4_]^+^ or in the tricoordinated cationic bis(nitrene) cobalt complex [(IMes)Co(NDipp)_2_]^+^.[^6^, ^14^] The latter low‐coordinated cationic complex is supported by the strongly electron‐donating and sterically demanding *N*‐heterocyclic carbene ligand IMes, and has been obtained by oxidation of the corresponding neutral bis(nitrene) Co^IV^ complex. Interestingly, theoretical calculations indicated that the frontier molecular orbitals of these bis(nitrene) complexes have near‐equal contributions from both the cobalt center and the nitrene ligand orbitals, indicating that the spectroscopic oxidation states for these cobalt centers are likely to be lower than IV and V, respectively. Apart from these bis(nitrene) complexes, the majority of the known cobalt nitrenoid complexes have low spin Co^III^ centers which are supported, for example, by bulky ancillary tripodal or bidentate ligands to achieve kinetic stabilization.^[15]^ To the contrary, terminal nitrido complexes of cobalt still remain elusive.^[16]^

## Results and Discussion

**Figure 1:**
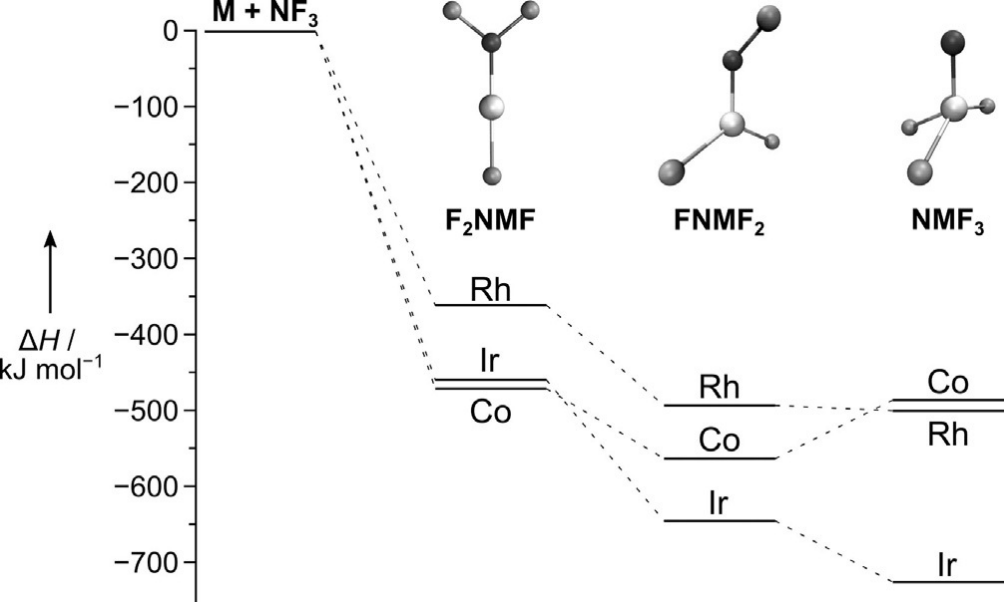
Stationary points on the reaction coordinate obtained at the B3LYP level of theory for the formation of the nitrido complexes N≡MF_3_ (M=Co, Rh, and Ir) from the free metal atoms M and NF_3_. See Table S1 for more details.

To obtain the group 9 metal difluorides, F−N=MF_2_ (M=Co, Rh, Ir), we have studied the gas‐phase reaction of the laser‐ablated free metal atoms with NF_3_ seeded in a 1:1000 excess of neon or argon. The reaction products were deposited on a gold‐plated copper mirror cooled to 5 and 12 K and IR‐spectroscopically investigated (for experimental details see the Supporting Information). According to preliminary calculations at the DFT‐B3LYP and BP86 levels of theory the direct insertion of the excited metal atoms into the F−N bond of NF_3_ to F_2_N−MF, and the subsequent fluorine migration from nitrogen to the metal center to yield the desired FN=MF_2_ is highly exothermic for all three metals (Figure 1, Table S1). However, the expected rearrangement of the fluoronitrene to a high‐valent nitrido trifluoride N≡MF_3_ is found to be endothermic for the cobalt complex, rendering FN=CoF_2_ the most stable CoF_3_N isomer. To the contrary, this rearrangement is slightly exothermic for the rhodium nitrene complex (Δ*H* ^0^=−12 kJ mol^−1^, CCSD(T)), and becomes strongly exothermic for the iridium congener (Δ*H* ^0^=−98 kJ mol^−1^, CCSD(T), Table S1), which rendered the detection of the iridium nitrene complex difficult if not impossible.

**Figure 2:**
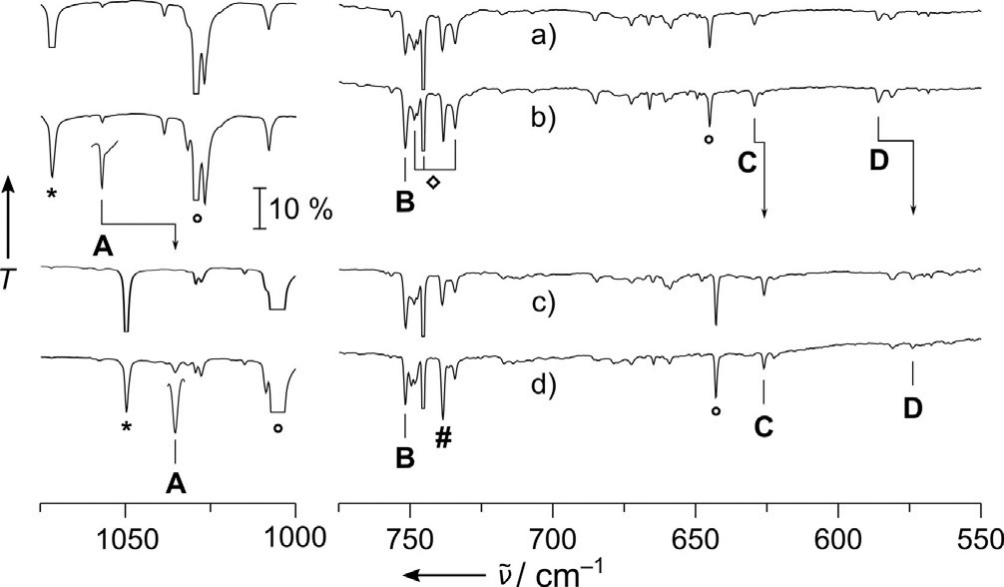
Infrared transmission spectra obtained after co‐depositing laser‐ablated cobalt atoms with 0.1 % ^14^NF_3_ in neon (a), with ^14^NF_3_ after annealing to 10 K (b) as well as with ^15^NF_3_ (c), with subsequent annealing to 10 K (d). Bands attributed to FNCoF_2_ are labeled **A**–**D**, and their ^14/15^N isotope shift is indicated. The cutout band labeled **A** is enhanced by factor 5. Further assignments are NF_2_ (asterisk), NF_3_ (circle) and CoF_*n*_ (square). The pound sign marks an unassigned product band.

**Figure 3:**
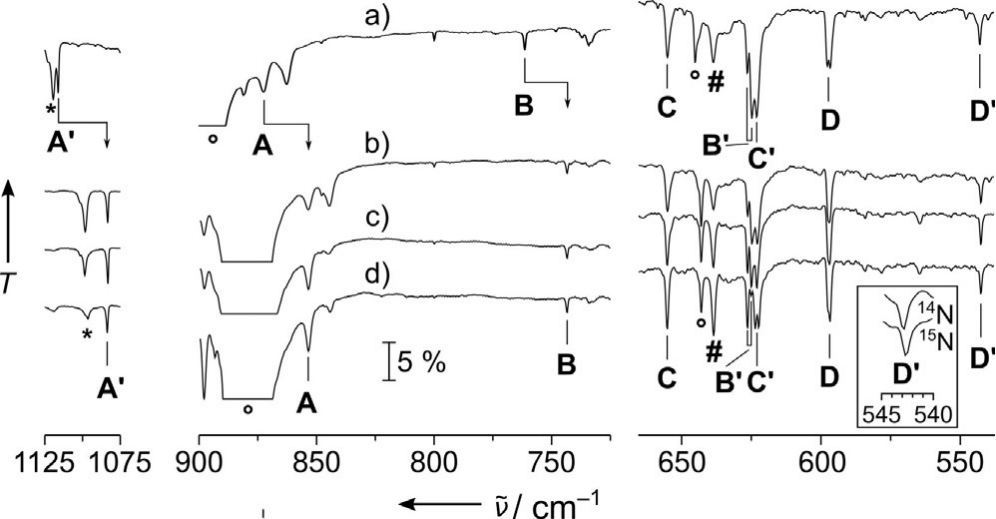
Infrared transmission spectra in the 1125–1075 cm^−1^ (left), 900–725 cm^−1^ (center) and 675–525 cm^−1^ (right) region from co‐depositing laser‐ablated rhodium atoms with 0.1 % ^14^NF_3_ in neon (a), and co‐depositing rhodium with ^15^NF_3_ in neon (b) with subsequent full‐arc photolysis (c) and annealing to 12 K (d). Bands attributed to FNRhF_2_ are labeled **A**–**D** and those assigned to NRhF_3_ are marked by **A′**–**D′**. Their ^14/15^N isotope shift is indicated. The enhanced inset shows the small ^14/15^N isotopic shift of **D′**. Further assignments are NF (asterisk) and NF_3_ (circle). The pound sign marks an unassigned product band which is the only band that gains intensity upon annealing.

**Figure 4:**
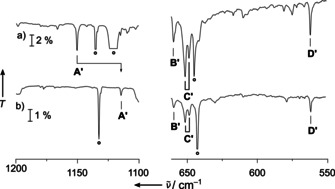
Infrared transmission spectra in the 1200–1100 cm^−1^ (left) and 630–550 cm^−1^ (right) region from co‐deposition of laser‐ablated iridium atoms with 0.1 % ^14^NF_3_ (a) and 0.1 % ^15^NF_3_ (b) in neon. Bands attributed to NIrF_3_ are labeled **A′**–**D′**, and their ^14/15^N isotope schift is indicated. Bands associated with NF_3_ are marked by a circle.

These predictions were fully supported by the analysis of the experimental IR spectra of the deposits in solid neon shown for cobalt (Figure 2), rhodium (Figure 3) and iridium (Figure 4). Complementary argon spectra for the experiments using rhodium and iridium have also been recorded and shown in the supporting information, Figures S1–S3. These spectra are dominated by strong bands of the NF_3_ precursor (Figure S4, Table S2 and Ref. [17]) and its plasma radiation induced decomposition products NF and NF_2_.^[18]^ However, the assignment of IR bands associated with the targeted nitrene and nitrido complexes is facilitated by a characteristic ^14/15^N isotope shift exhibited by all modes in which the nitrogen atom is significantly involved. These isotope shifts are indicated in the experimental spectra shown in the Figures 2–4. They were obtained in experiments using ^15^NF_3_, which was synthesized from ^15^N_2_ and F_2_ mixtures in an electric discharge.^[19]^ While a detailed report about the spectral assignment is given in the Supporting Information, it should be mentioned here, that bands due to binary metal fluorides MF_*n*_ also appeared in these spectra, however these were safely assigned in nitrogen‐free experiments, in which NF_3_ was replaced by elemental fluorine. In these experiments none of the bands assigned to a nitrogen‐containing species appeared. Furthermore, by comparing spectra of experiments using different group 9 metals, the desired metal dependent bands were identified. A list of all observed IR bands associated with the target compound is shown in Table 1 together with their approximate assignment and supporting predictions from quantum‐chemical calculations.

**Table 1:** Comparison of infrared band positions (cm^−1^) and isotopic shifts (cm^−1^, in parenthesis) observed in solid neon and argon with calculated values [intensities in km mol^−1^ in brackets] and their assignment in terms of an approximate description of the vibrational modes.^[a]^

Neon	Argon	BP86	B3LYP	CCSD(T)	Assignment
FNCoF_2_					
1056.8 (−21.4)	–^[b]^	870 (−19) [220]	1080 (−20) [293]	1152 (−22) [–] ^[c]^	F−N str. [a′]
751.7 (−0.1)	–^[b]^	732 (0) [123]	764 (0) [171]	859 (−0.3)[–]^[c]^	antisym. F−Co−F str. [a′′]
629.6 (−3.5)	–^[b]^	627 (−1) [63]	611 (0) [60]	651 (−1) [–]^[c]^	sym. F−Co−F str. [a′]
586.1 (−12.1)	–^[b]^	765 (−17) [37]	442 (−13) [12]	609 (−16) [–] ^[c]^	N−Co str. [a′]

NRhF_3_					
1116.1 (−33.0)	1112.56 (−33)	1087 (−32) [48]	1113 (−33) [55]	–	N−Rh str. [a′]
626.2/ 624.8 (0)^[d]^	612.0	603 (0) [96]	625 (0) [118]	–	antisym. F−Rh−F str. [a′′]
622.2/ 622.8 (0)^[d]^	610.5	601 (0) [48]	618 (0) [63]	–	sym. F−Rh−F str. [a′]
542.5 (−0.4)	539.9 (−0.5)	562 (0) [45]	581 (0) [52]	–	Rh−F′ str. [a′]

FNRhF_2_^[e]^					
872.6 (−18.9)	–^[b]^	850 (−24) [103]	935 (−24) [168]	872 (−16) [–]	F−N str. [a′]
761.4 (−18.0)	760.1 (−19.6)	721 (−12) [181]	786 (−16) [117]	707 (−19) [–]	N−Rh str. [a′]
655.1 (0)	638.9 (0)	625 (0) [132]	641 (0) [150]	652 (0) [–]	antisym. F−Rh−F str. [a′′]
596.7 (0)	585.9 (−0.5)	572 (−0.3) [101]	594 (−0.3) [75]	580 (−2) [–]	sym. F−Rh−F str. [a′]

NIrF_3_					
1150.4 (−36.0)	1144.6 (−35.8) ^[f]^	1121 (−35) [22]	1158 (−36) [23]	1126 (−36) [–]	N−Ir str. [a′]
659.8 (0)	–^[g]^	618 (0) [34]	635 (0) [43]	653 (0) [–]	sym. F−Ir−F str. [a′]
651.6/648.9 (0)^[c]^	–^[g]^	618 (0) [132]	634 (0) [147]	650 (0) [–]	antisym. F−Ir−F str. [a′′]
562.1 (−0.2)	560.1 (−0.5)^[f]^	562 (0) [36]	581 (0) [40]	607 (0) [–]	Ir−F′ str. [a′]

[a] Only normal modes predicted in the experimentally observable range ($\tilde{\nu }>$ 400 cm^−1^) are listed. A full list of computed frequencies is presented in the Supporting Information. For CASPT2 and CCSD(T) no intensities are available; [b] Bands not observed, or too weak. [c] Wavenumbers (isotopic shifts) obtained at the CASPT2/cc‐pVTZ‐DK level; [d] Two matrix sites; [e] Wavenumbers (isotopic shifts, in cm^−1^) obtained using CASPT2/cc‐pVTZ‐DK: 981 (−19.5), 756 (−20.4), 672 (0), 623 (−0.5); [f] Weak bands tentatively assigned. [g] Too weak or overlapped by broad and strong NF_3_ bands in this area.

**Figure 5:**
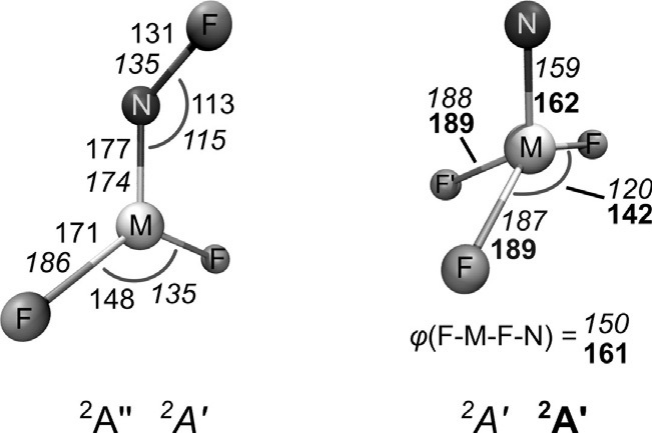
Electronic ground state structures of FNMF_2_ for M=Co (^2^A′′, regular) and Rh (^2^A′, italic) calculated at the CASPT2/cc‐pVTZ‐DK level and of NMF_3_ for M=Rh (^2^A′, italic) and Ir (^2^A′, bold) obtained at the CCSD(T)/aug‐cc‐pVTZ(‐PP) level of theory. Bond lengths are given in pm and angles in degree (*ϕ* denotes the dihedral angle of F‐M‐F‐N).

In the experiment using laser‐ablated Co atoms and NF_3_ four IR bands were obtained that displayed a characteristic ^14/15^N isotope shift (labeled **A**–**D** in Figure 2) and their assignment to the targeted fluoronitrene complex FNCoF_2_ is well supported by prediction on the CASPT2/cc‐pVTZ‐DK level of theory (Table 1). The values obtained using single reference correlation methods did either not converge (CCSD(T)), or did not yield qualitatively consistent results (B3LYP and BP86). Bands associated to the desired nitrido complex NCoF_3_ were not detected, which is consistent with the significant higher energy of this isomer. On the other side, in the spectra obtained from laser‐ablated Ir atoms and NF_3_ our search for bands due to FNIrF_2_ was unsuccessful and only the nitrido complex NIrF_3_ was formed. Again, this reflects the lower stability of the former species, which exothermically rearranged to the lowest energy isomer. Here, too, four bands were assigned to NIrF_3_ (marked with **A′**–**D′** in Figure 4), of which only two revealed a ^14/15^N isotope shift. Quantum‐chemical calculations (Table 1) performed at the DFT (BP86, B3LYP) and CCSD(T) levels of theory fully support these assignments. The IrF_3_ stretching modes are split into three components due to a first order Jahn–Teller distortion for the anticipated 5*d* ^3^ configuration, which reduced the full *C*_3*v*_ point group symmetry to *C*_s_ symmetry (for structures see Figures 5, S5, and Table S3). All three Ir−F stretching modes were observed, but only the band associated with the Ir−F′ bond, which resides in the mirror plane together with the N−Ir bond, show a small ^14/15^N isotope shift.

**Figure 6:**
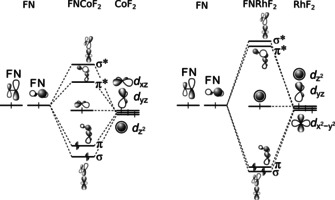
Simplified MO diagrams for the leading electronic configurations of the 4‐electron‐3‐center F‐N‐M bond of FNCoF_2_ (left) and FNRhF_2_ (right). The low‐energy F−N bonding orbitals are not shown.

The assignment of the IR spectra obtained after co‐depositing evaporated rhodium and diluted NF_3_ (Figure 3) was more puzzling. In these experiments both the anticipated compounds are finally detected in the solid matrices and for each all four stretching bands were successfully assigned (Table 1). As described above for the corresponding Iridium compound also for NRhF_3_ a ^14/15^N isotope shift was observed for the N−Rh and the Rh−F′ stretching modes (Figure 3, **A′** and **D′**, respectively). Our CCSD(T) calculations for this species yield two imaginary frequencies which likely are caused by a close‐lying excited electronic state which interferes with the calculation of displaced steps during the numerical hessian calculation where the symmetry is lowered to *C*_1_. However, the structure obtained at the B3LYP level of theory is close to the one obtained at the CCSD(T) level (Figures 5 and S5) and the good agreement of the B3LYP results for NIrF_3_ with the experimental frequencies suggest a good performance also for the NRhF_3_ species. The ^14/15^N isotope shift observed for the nitrene complex FNRhF_2_ is well distributed between the F−N and the N−Rh stretching bands (Figure 3, bands **A** and **B**, respectively) indicating a strong vibrational coupling between these two modes. Analyzing the N−M and F−N stretching frequencies of FNCoF_2_ and FNRhF_2_ we found surprisingly large differences in the bonding of the fluoronitrene ligand. In general, the two singly occupied anti bonding π*(F−N)‐orbitals of the FN ligand form a σ and a π bond to these metal centers, as depicted in the qualitative molecular orbital (MO) interaction diagram shown in Figure 6. The F−N mode of FNCoF_2_ (1056.8 cm^−1^) appeared red‐shifted by 62.6 cm^−1^ from the absorption of free, neutral FN (1119.4 cm^−1^),^[11]^ indicating the presence of an almost neutral nitrene ligand, while the corresponding mode of FNRhF_2_ (872.6 cm^−1^) is much stronger red‐shifted by 246.8 cm^−1^.

On the other side, the low N−Co stretching frequency of FNCoF_2_ (586.1 cm^−1^) is most likely associated with a N−Co single bond, while the N−Rh frequency of FNRhF_2_ (761.4 cm^−1^) can be attributed to a N=Rh double bond. These experimental observations are consistent with calculated atomic charges for the FN fragment in FNCoF_2_ (NPA: 0.02, AIM: −0.02) and FNRhF_2_ (NPA: −0.25, AIM: −0.20), as well as Mayer and Wiberg bond orders for the N−M bonds listed in Table 2, and the calculated N−M bond lengths (M=Co: 177 pm, Rh: 174 pm, Figure 5). To shed light on these striking bonding differences non‐dynamical electron‐correlation effects were taken into account. The results of CASSCF calculations revealed that the leading configuration (σ^2^π^2^δ^1^ π*^0^σ*^0^) associated with the qualitative MO Scheme shown in Figure 6 contributes only 48 % to the ^2^A′′ ground state of FNCoF_2_, followed by states with significant weights which contain single and double π→π* excitations (Table S4). For FNRhF_2_ a much smaller extend of non‐dynamic correlation was determined, since the most dominant configuration as depicted in Figure 6 contributes to 84 % to its ^2^A′ electronic ground state. As a consequence of these correlation effects significant higher σ* and π* populations (0.37 and 0.61, Figure S6) were found for FNCoF_2_ compared to FNRhF_2_ (σ*: 0.11, π*: 0.18). The effective bond orders (EBO)^[20]^ derived from the natural orbitals obtained at the CASSCF level are 1.1 for the Co−N and 1.7 for the Rh=N bond. We also note a considerable amount of minority spin population at the N atom (−0.46) in FNCoF_2_ (Table 2 and Figure S7) antiferromagnetically coupled to the majority spin at the Co center (1.46). The latter spin density can mainly be attributed to the singly occupied nonbonding δ‐MO of a′′ symmetry (Figures 6 and S6). Taking these effects into account, the FN unit in FNCoF_2_ has at least a considerable fraction of iminyl/nitrene radical character, which explains the shortened single bond. The disparities in the metal‐nitrogen bonds of these nitrene complexes can likely be attributed to the peculiarity of bonding of the strongly correlated first‐row transition metal‐ligand bonds. Especially the close internuclear distance required for an optimum orbital overlap for π bonding is likely hindered due to Pauli repulsion of the Co 3s,3p core–shell and the nitrogen ligand orbitals.^[21]^

**Table 2:** NPA and AIM charges, spin populations, as well as Mayer and Wiberg bond orders obtained from electronic ground state wavefunctions calculated at the CASSCF(9,7)/cc‐pVTZ‐DK levels of theory. All units in atomic units.

Property		F′NCoF_2_ (^2^A′′, *C*_s_)	F′NRhF_2_ (^2^A′, *C*_s_)
NPA Charge	F′	−0.2342	−0.2508
	N	0.2552	0.1196
	M	1.6134	1.6264
	F	−0.8172	−0.7476
			
AIM Charge	F′	−0.3780	−0.3932
	N	0.3576	0.1916
	M	1.6158	1.6929
	F	−0.7977	−0.7457
			
Spin Population	F′	−0.0239	−0.0006
	N	−0.4639	−0.1149
	M	1.4593	1.0627
	F	0.0143	0.0264
			
Mayer Bond Order	F′‐N	0.976	0.909
	N‐M	0.561	1.395
	M‐F	0.647	0.647
			
Wiberg Bond Order	F′‐N	1.429	1.337
	N‐M	1.246	2.156
	M‐F	1.180	1.167

For the nitrido complexes NRhF_3_ and NIrF_3_ the computed bond length (162 pm (IrN), 159 (RhN); for comparison: triple‐bond additive covalent radii: 160 pm (IrN) and 160 pm (RhN),^[22]^ Figure 5) and the experimental stretching frequencies (Table 1) indicate strong N≡M triple bonds. An analysis of the CASSCF(9,8) natural molecular orbitals (Figure S8) reveals EBOs of 2.7 and 2.8 for N≡Rh and N≡Ir, respectively. Consistent with the assignment of oxidation state +VI for both metal centers, a *d* ^3^ configuration and a Jahn–Teller distorted ^2^A′ electronic ground state was determined for both species. The N−Ir stretching frequency in neon of 1150.4 cm^−1^ is moderately higher than that observed for diatomic IrN embedded in solid neon (1111.1 cm^−1^).^[23]^ However, in case of rhodium a significant blue‐shift of the N−Rh stretching mode (1112.6 cm^−1^) of 221.2 cm^−1^ occurred compared to diatomic RhN embedded in argon (891.4 cm^−1^).^[24]^ The increased force constant of 920 N m^−1^ in NRhF_3_ from 580 N m^−1^ in RhN indicates a significant strengthening of the nitrogen‐metal bond induced by the fluorine ligands. This fluorine effect is less pronounced for the already strong triple bond in IrN, where force constants increase from 950 to 1020 N m^−1^ for IrN and NIrF_3_, respectively.

## Conclusion

In summary, we described the exothermic formation of the fluoronitrenoid complexes FNCoF_2_ and FNRhF_2_ and of the nitrido complexes NRhF_3_ and NIrF_3_ by the reaction of the free group 9 metal atoms with NF_3_. The IR spectra of these compounds isolated in solid rare gases (neon and argon) were recorded and, aided by the observed ^14/15^N isotope shifts and quantum‐chemical predictions, all four stretching fundamentals of these complexes were safely assigned. Neither nitrido nor other ternary complexes of Rh^VI^ and Ir^VI^ have yet been reported. The anticipated rearrangement of FNCoF_2_ to the nitrido Co^VI^ complex was not observed, because this reaction is endotherm. The covalently bound FN ligand in these high‐valent metal complexes is almost neutrally charged, and the formal picture of an FN^2−^ ligand bound to a MF_2_ fragment is a rather coarse approximation for these fluoronitrenoid complexes. However, the bonding of the FN ligand in FNCoF_2_ and FNRhF_2_ was found to be strikingly different. In FNCoF_2_ the FN ligand is singly bonded to Co and bears considerable iminyl/nitrene radical character, while the N=Rh double bond in FNRhF_2_ shows comparatively strong σ‐ and π‐bonds. The stretched N‐Co bond and the poor overlap especially between the ligand π‐ and the metal 3d‐orbitals can likely be attributed to a repulsion between the ligand orbitals and the outermost core 3s,3p shell of cobalt.

## Conflict of interest

The authors declare no conflict of interest.

## Supporting information

As a service to our authors and readers, this journal provides supporting information supplied by the authors. Such materials are peer reviewed and may be re‐organized for online delivery, but are not copy‐edited or typeset. Technical support issues arising from supporting information (other than missing files) should be addressed to the authors.

Supplementary
