# Supplementary material for: Fluoro Nitrenoid Complexes FN=MF2 (M=Co, Rh, Ir): Electronic Structure Dichotomy and Formation of Nitrido Fluorides N≡MF3
Source: Angew Chem Int Ed Engl. 2020 Oct 15;59(51):23174–9. doi: 10.1002/anie.202010950 (PMC7756499; doi:10.1002/anie.202010950)
Supplement: Supplementary file 1 — Supplementary [file ANIE-59-23174-s001.pdf]

Angewandte Chemie
Eine Zeitschrift der Gesellschaft Deutscher Chemiker

Supporting Information

Fluoro Nitrenoid Complexes $FN{=}MF_2$ ($M = Co, Rh, Ir$): Electronic Structure Dichotomy and Formation of Nitrido Fluorides $N{\equiv}MF_3$

*Tony Stüker, Thomas Hohmann, Helmut Beckers, and Sebastian Riedel**

anie_202010950_sm_miscellaneous_information.pdf

Contents

Contents 1

Experimental and Computational Details 1

Detailed Assignments 2

Synthesis of $^{15}NF_3$ 4

Supporting Figures 6

Supporting Tables 13

Detailed Computational Results 15

Supporting Information References 34

Experimental and Computational Details

Computational Details

Density functional theory (DFT) calculations were performed using the TURBOMOLE 7.0.1 program package[1] employing the GGA and hybrid exchange-correlation density functionals BP86[2] and B3LYP[3] with the polarized quadruple-ξ basis set def2-QZVP[4] which applies the Stuttgart-Dresden effective core potential for rhodium and iridium[5]. The Coupled Cluster Single Double and perturbative Triple excitations (CCSD(T)) calculations were carried out in the spin unrestricted ROHF-UCCSD(T) open-shell coupled cluster formalism using default frozen core settings as implemented in the Molpro 2019 software package.[6] The same software was used for all Complete Active Space Self Consistent Field (CASSCF) and Complete Active Space Perturbation Theory Second Order (CASPT2) calculations. Unless stated otherwise, all CCSD(T) calculations were combined with the augmented triple-ξ basis sets aug-cc-pVTZ for nitrogen and fluorine, and aug-cc-pVTZ-PP for rhodium and iridium.[7–9] CASPT2 calculations for $FNRhF_2$ and $FNCoF_2$ were carried out with relativistic corrections using the second order Douglas-Kroll-Hess Hamiltonian combined with the Dunning's correlation consistent polarized triple-ξ basis sets cc-pVTZ-DK[7,8,10]. The active space for the state-specific complete active-space (SS-CASSCF) reference wavefunction was chosen to consist of the M-N σ and π binding and anti-binding and the metal centered d-type molecular orbitals formed by the 2p(N), 3d(Co) and 4d(Rh) atomic orbitals, yielding 9 electrons in 7 molecular orbitals (9,7) for $FNCoF_2$ and $FNRhF_2$. Single point SS-CASSCF calculations carried out for $NRhF_3$ and $NIrF_3$ consisted of an active space of of 9 electrons in 8 orbitals covering the M-N three bonding, three antibonding and the singly and doubly occupied metal centered molecular orbitals. The lowest 9 molecular orbitals for $FNCoF_2$/$NCoF_3$ and the lowest 18 molecular orbitals for $FNRhF_2$/$NRhF_3$ were frozen in the subsequent CASPT2 dynamic

correlation treatment. Harmonic vibrational frequency calculations were carried out for optimized structures analytically (BP86, B3LYP) or numerically (CCSD(T) and CASPT2).Matrix-isolation Experiments

$^{14}NF_3$ and $^{15}NF_3$ (vide infra) were premixed with neon or argon (both 99.999 %, Linde) in a stainless-steel cylinder. The mixing vessel was connected to a stainless-steel vacuum line connected to a self-made matrix chamber by a stainless-steel capillary. The gas mixture was then co-deposited for 100 min with laser-ablated cobalt, rhodium or iridium atoms onto a CsI window (argon matrices) or onto a gold plated copper mirror (neon matrices) and cooled to 4 K by using a closed-cycle helium cryostat (Sumitomo Heavy Industries, RDK–205D) inside the vacuum chamber. For the laser-ablation, the 1064 nm fundamental of a Nd:YAG laser (Continuum, Minilite II, 10 Hz repetition rate, 35–50 mJ pulse^{-1}) was focused onto a rotating iridium metal target through a hole in the cold window. Infrared spectra were recorded on a Bruker Vertex 70 spectrometer purged with dry air (argon matrices) or a Bruker Vertex 80v with evacuated optical path (neon matrices) at 0.5 cm^{-1} resolution in the region 4000–430 cm^{-1} by using a liquid-nitrogen-cooled mercury cadmium telluride (MCT) detector. The matrix samples were irradiated by a mercury arc streetlamp (Osram HQL 250) with the outer globe removed.

Detailed Assignments

Laser-ablated iridium, rhodium and cobalt atoms were reacted with $^{14}NF_3$ and $^{15}NF_3$ in a 1:1000 excess of neon or argon and deposited on a gold-plated copper mirror cooled to 5 and 12 K, respectively. The IR spectra in excess neon are shown in Figures 2–4 for iridium, rhodium and cobalt. The complementary argon spectra for iridium and rhodium are shown in Figures S1-S3. By comparing spectra of the reaction products of one metal with those of another metal, the metal dependent bands were identified. Further simplification was achieved by neglecting bands belonging to MF_n obtained by recording complementary spectra of metal fluorine reaction products under the same conditions. It is worth pointing out that the IR spectra obtained in neon are generally of better quality in terms of intensity and line broadening. This is because neon is being considered the least interacting and most inert cryogenic matrix available.[11]

$FNCoF_2$

After co-depositing laser-ablated Co and NF_3 diluted in Ne, the IR spectra shown in Figure S11 and S12 were recorded. The 1100–725 cm^{-1} region in Figure S11 contains two bands. A weak band at 1056.8 cm^{-1} which exhibits an $^{14/15}$N isotopic shift of −21.4 cm^{-1} and at 751.7 cm^{-1} without isotopic shift. In the spectral region of 725–525 cm^{-1} shown in Figure S12 two bands with an isotopic shift are located at 629.6 and 586.1 cm^{-1} with isotopic shifts of −3.5 and −12.1 cm^{-1}, respectively. The isotopic shift of the band at 1056.8 cm^{-1} is considereably less than expected for a terminal bond nitrogen atom and consistent with an assignment to to the F-N stretching mode of $FNCoF_2$. The remaining bands at 751.7, 629.6 and 586.1 cm^{-1} are assigned to the antisymmetric F-Co-F, symmetric F-Co-F and to the N-Co stretching mode. Attempts to use single reference correlation methods to calculate the geometry and frequencies of this species failed. They did not converge (CCSD(T)), or did not yield qualitatively

consistent results (B3LYP and BP86). To account for the strong non-dynamical correlation, CASSCF(9,7) with subsquent CASPT2 dynamical correlation treatment was employed. The values calculated at the CASPT2/cc-pVTZ-DK level of theory are in good agreement with the experiment (Table 1). The assignment of $FNCoF_2$ is further supported by the fact that the lowest energy structural isomer based on the reaction enthalpies summarized in Table S1.

$NRhF_3$

The IR spectra obtained after co-depositing evaporated rhodium and diluted NF_3 in neon shown in Figures S13 and S14 contain three bands with significant isotopic shifts. The position of the band at 1116.1 cm^{-1} (Figure 3, **A**) and the isotopic shift of −33 cm^{-1} are indicative for a terminally bond nitrogen with a triple bond. This band, as well as three additional bands in the M-F stretching region were assigned to $NRhF_3$. The bands located at 626.2 and 622.8 cm^{-1} loose intensity upon broadband irradiation and annealing to 12 K with their initially weaker matrix sites at 624.8 and 622.2 cm^{-1} gaining intensity. These bands were assigned to the antisymmetric and symmetric F-Rh-F stretching mode, respectively. The last band is located at 542.5 cm^{-1} shows a small isotopic shift of −0.5 cm^{-1} and was assigned to the Rh-F' stretching mode. The assignments are supported by quantum chemical calculations, which yield 1087 (1113), 625 (603), 618 (601) and 581 (562) cm^{-1} at the BP86 (B3LYP) levels of theory. The calculated isotopic shift of the N-Rh stretching mode is −32 (−33) at the BP86 (B3LYP) level of theory, respectively, and in very good agreement with the experimentally observed values in neon and in argon (both −33 cm^{-1}). The small observed isotopic shift of −0.4 (−0.5) cm^{-1} of the Rh-F' stretching mode observed in neon (argon) was not predicted by both DFT methods but is also observed in $NIrF_3$ as well. Unfortunately, calculations at the CCSD(T) level of theory yield two imaginary frequencies probably caused by a low-laying excited electronic state which interferes with the calculation of displaced steps during the numerical hessian calculation where the symmetry is lowered to C_1. However, the structure obtained at the B3LYP level of theory is very close to the one obtained at the CCSD(T) level (Figures 5 and S5) and the good match of the B3LYP results for $NIrF_3$ with the experimental values suggest a good performance of B3LYP for the NMF_3 species.

$FNRhF_2$

The two remaining bands with significant isotopic shifts of −18.9 and −18.0 cm^{-1} (labeled **B** and **C** in Figure S13) centered at 872.6 and 761.4 cm^{-1} are indicative for absorptions of two modes exhibiting significant displacements of a nitrogen atom which is not terminally bonded. The assignment to the two modes involving the F-N-Rh moiety of $FNRhF_2$ suggests itself, however, while quantum chemical calculations at the DFT BP86 (B3LYP) level of theory yield acceptable band positions of 850 (935) and 721 (786) cm^{-1}, the isotopic shifts of −24 (−24) and −12 (−16) cm^{-1} do not match the observed ones. More sophisticated calculations at the CCSD(T) (CASPT2) levels of theory yield isotopic shifts of −16 (−19) and −19 (−20) (Table S5) which are in better agreement with the observed values. However, the deviations between the calculated and observed band positions at 899 (981) and 720 (756) cm^{-1} indicates the presence of vibronic coupling effects which are not taken into account in the harmonic approximation. The band centered at 872.6 cm^{-1} was assigned to the F-N stretching mode, while the band at 761.4 cm^{-1} was assigned to the N-Rh stretching mode. Along with vibrations related to the F-

N-Rh moiety, the bands belonging to the antisymmetric and symmetric F-Rh-F stretching modes were assigned to bands at 638.6 and 596.7 cm^{-1} (labeled **A** and **D** in Figure S14) in an overall very good agreement with the calculated values summarized in Table 1. The antisymmetric F-N-Rh stretching mode could not be identified in the corresponding argon spectrum. Additional bands are much weaker. They are tentatively assigned in Figures S1 and S2 and summarized in Table 1. The presence of both species, $NRhF_3$ and $FNRhF_2$, is reasonably justified considering the small energy difference of 7 [12] kJ mol^{-1} calculated at the B3LYP [CCSD(T)] level of theory (see Table S1 and Figure 1).

$NIrF_3$

After depositing iridium and NF_3 diluted in neon, bands immune to annealing and broadband irradiation centered at 1150.4, 659.8, 651.6, 648.9 and 562.1 cm^{-1} shown in Figure S15 were singled out and assigned to $NIrF_3$ with a point group symmetry of C_s. The position of the band centered at 1150.4 cm^{-1} (Figure S15, **A**), the isotopic shift of −36 cm^{-1} and the $^{14/15}N$ isotopic ratio of 1.0323 are indicative for a vibration of a strongly bond single nitrogen atom attached to a heavy element. Theoretical calculations support the assignment to the Ir≡N stretching mode, with calculated isotopic shifts of −35, −36, and −36 and $^{14/15}N$ isotopic ratios of 1.0325, 1.0325 and 1.0326 obtained the BP86/def2-QZVP, B3LYP/def2-QZVP and ROHF-UCCSD(T)/aug-cc-pVTZ(-PP) levels of theory, respectively. However, the absolute values of 1121, 1158 and 1126 cm^{-1} (BP86, B3LYP and CCSD(T)) deviate by −29, 8 and −24 cm^{-1}, respectively. The assignment of the absorption at 659.8 cm^{-1} (Figure S15, **B**) to the symmetric F-Ir-F stretching mode is backed by calculated values of 618, 635 and 653 cm^{-1} (BP86, B3LYP and CCSD(T)). Isotopic shifts were not observed and were calculated to be 0 for the $^{14/15}N$ substituted isotopologue. The more intense antisymmetric F-Ir-F stretching mode and its matrix site are located at 651.6 and 648.9 cm^{-1} (Figure S15, **C**) and do not show any matrix shift either. The calculated values of 618, 634, and 650 cm^{-1} (BP86, B3LYP, CCSD(T)) confirm the proximity of the symmetric and antisymmetric F-Ir-F stretching frequencies. Finally, the band showing a very small isotopic shift of −0.2 cm^{-1} centered at 562.1 cm^{-1} (Figure S15, **D**) was assigned to the F'-Ir stretching mode involving the fluorine atom residing the mirror plane of the molecule. The IR spectra obtained in argon (Figure S3) are not as comprehensive as in neon. The Ir-N stretching mode and the F'-Ir stretching mode were tentatively assigned to very weak bands centered at 1144.6 and 560.1 cm^{-1}, respectively. The bands belonging to the symmetric and antisymmetric F-Ir-F stretching modes are probably overlapped by the bands belonging to the strong and broad symmetric deformation mode of NF_3 and its matrix sites.

Synthesis of $^{15}NF_3$

The synthesis of $^{15}NF_3$ was carried out in an electric discharge cell described by Ruff and Menzel[12] following the procedure described by Maya[13]. A 1:4 mixture of $^{15}N_2$ and F_2 was slowly allowed to enter an electric discharge cell submerged in liquid nitrogen (LN_2) shown in Figure S9. The electronic discharge arc was generated by a current of 7.0 kV, while the flow was adjusted to maintain a constant pressure of 30–35 mbar inside the reactor. After 1.5 h a volume of 113 mL of the $^{15}N_2/F_2$ mixture was admitted to the reactor. The condensed, crude reaction product at the bottom of the reactor was

recondensed into a glass cylinder. The volatile gas phase of the LN2 cooled crude phase contained F_2 (identified through the presence of SiF_4). To remove all remaining F_2 from the crude product, the glass cylinder was cooled using a N_2 slurry and pumping off all volatile components for 5 min at 10^{-3} mbar. The purification process was followed by gas phase IR spectra shown in Figure S0. The purified product did not show any IR active impurities in a matrix-isolation spectrum in 99.9 % excess neon shown in Figure S4. In addition to the assignments by Allan et al.[14], six more combination- and overtone bands were identified and listed in Table S2. The overall yield was 73 % with respect to $^{15}N_2$, significantly higher than the reported yield of 30 % $^{14}NF_3$.[13]

Supporting Figures

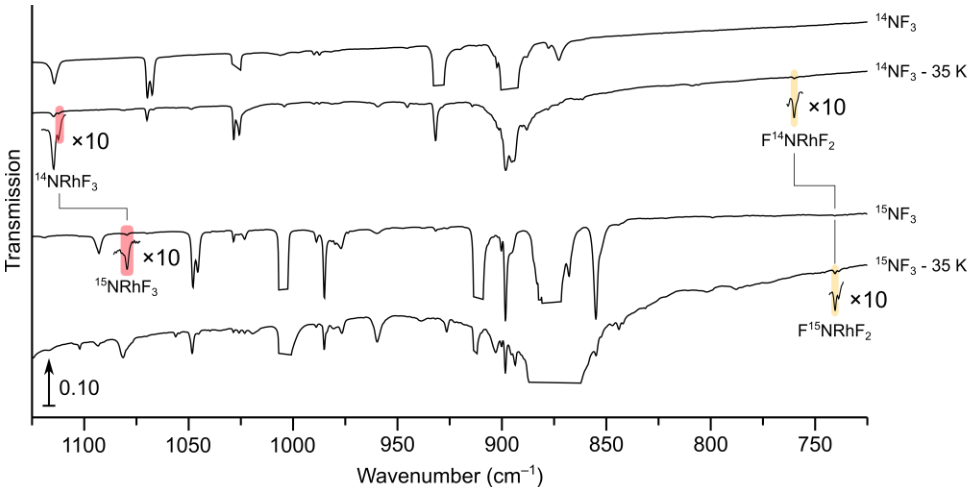

Figure S1. Infrared transmission spectra in the 1125–725 cm^{-1} region of laser ablated rhodium co-deposited with 0.5 % $^{14}NF_3$ and $^{15}NF_3$ diluted in argon and treated according to the labels.

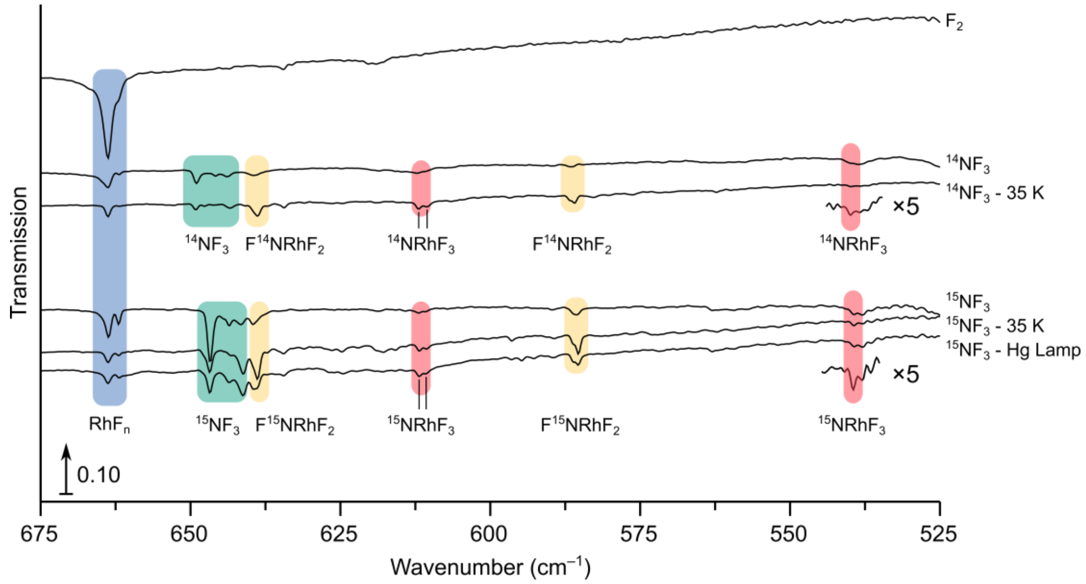

Figure S2. Infrared transmission spectra in the 675–525 cm^{-1} region of laser ablated rhodium co-deposited with 0.5 % of F_2, $^{14}NF_3$ and $^{15}NF_3$ diluted in argon and treated according to the labels.

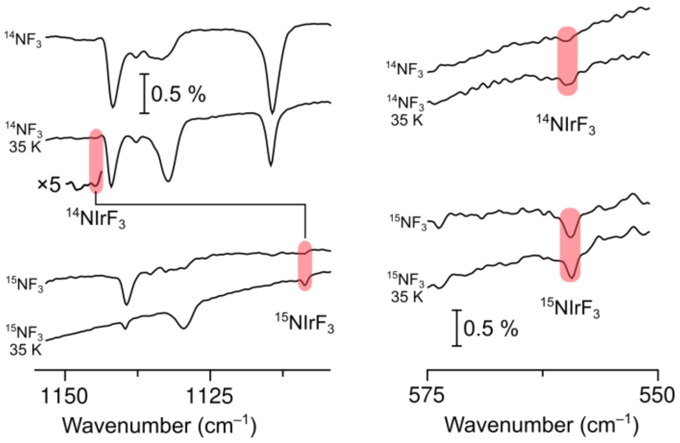

Figure S3. Infrared transmission spectra in the 1155–1100 and 575–550 cm^{-1} regions of laser ablated iridium co-deposited with 0.5 % $^{14}NF_3$ and $^{15}NF_3$ diluted in argon and treated according to the labels.

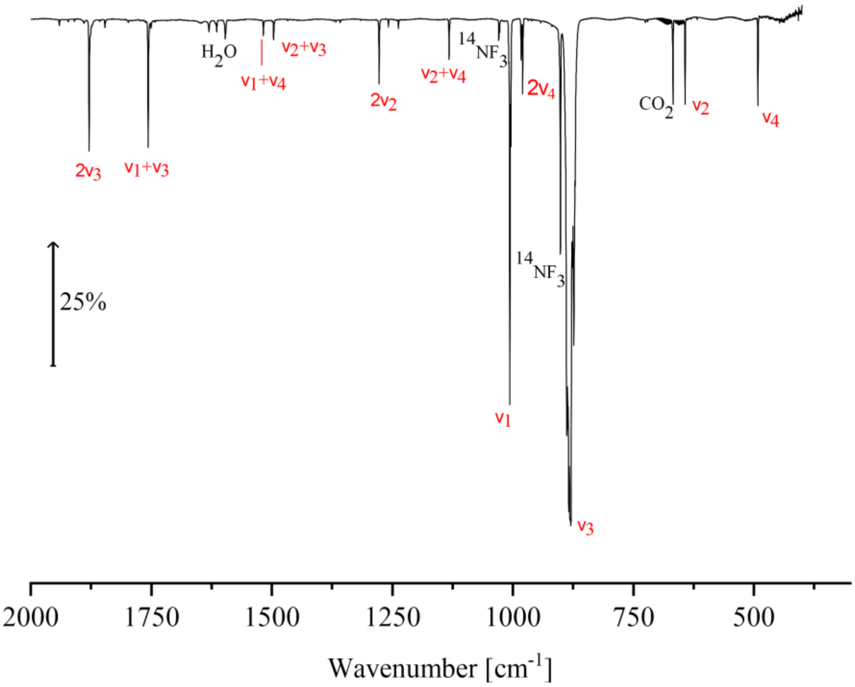

Figure S4. IR spectrum of $^{15}NF_3$ in excess neon (0.1%) at 4.2 K.

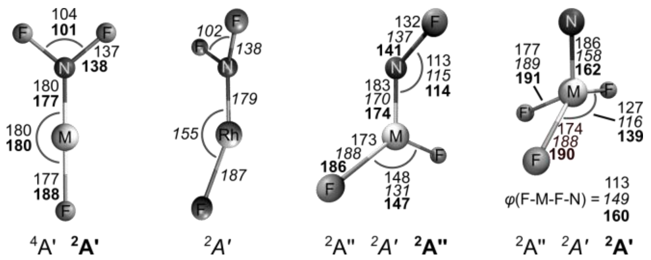

Figure S5. Molecular structures and electronic ground state symbols calculated at the B3LYP level of theory. Bond lengths in pm and angles in degree (φ denotes the dihedral angle of F-M-F-N). Values for M = Co (normal), Rh (italic) and Ir (bold).

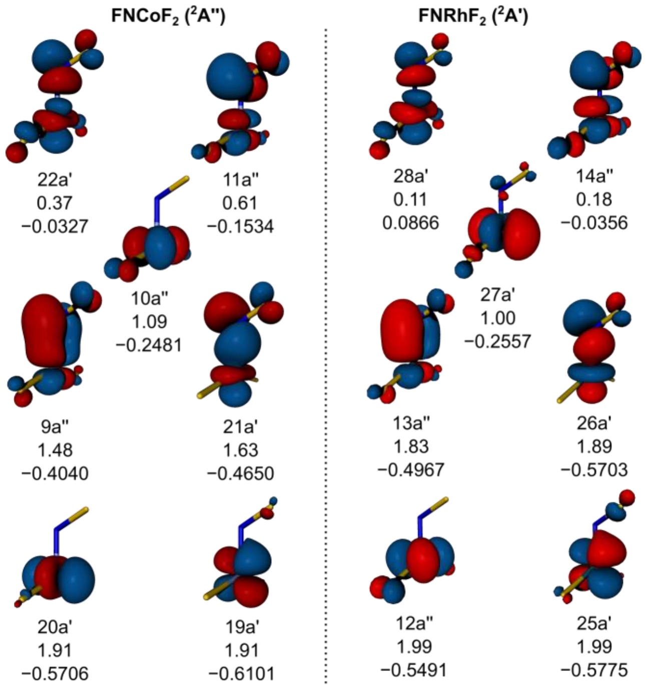

Figure S6. Natural frontier molecular orbitals (isovalue 0.05 a.u.) of $FNCoF_2$ (left) and $FNRhF_2$ (right) calculated at the CASSCF(9,7)/cc-pVTZ-DK level of theory, including irreps, occupation numbers and orbital energies.

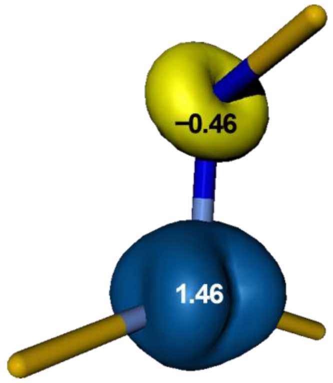

Figure S7. Spin density plot (isovalue 0.03 a.u.) of $FNCoF_2$ calculated at the CASSCF(9,7)/cc-pVTZ-DK level of theory.

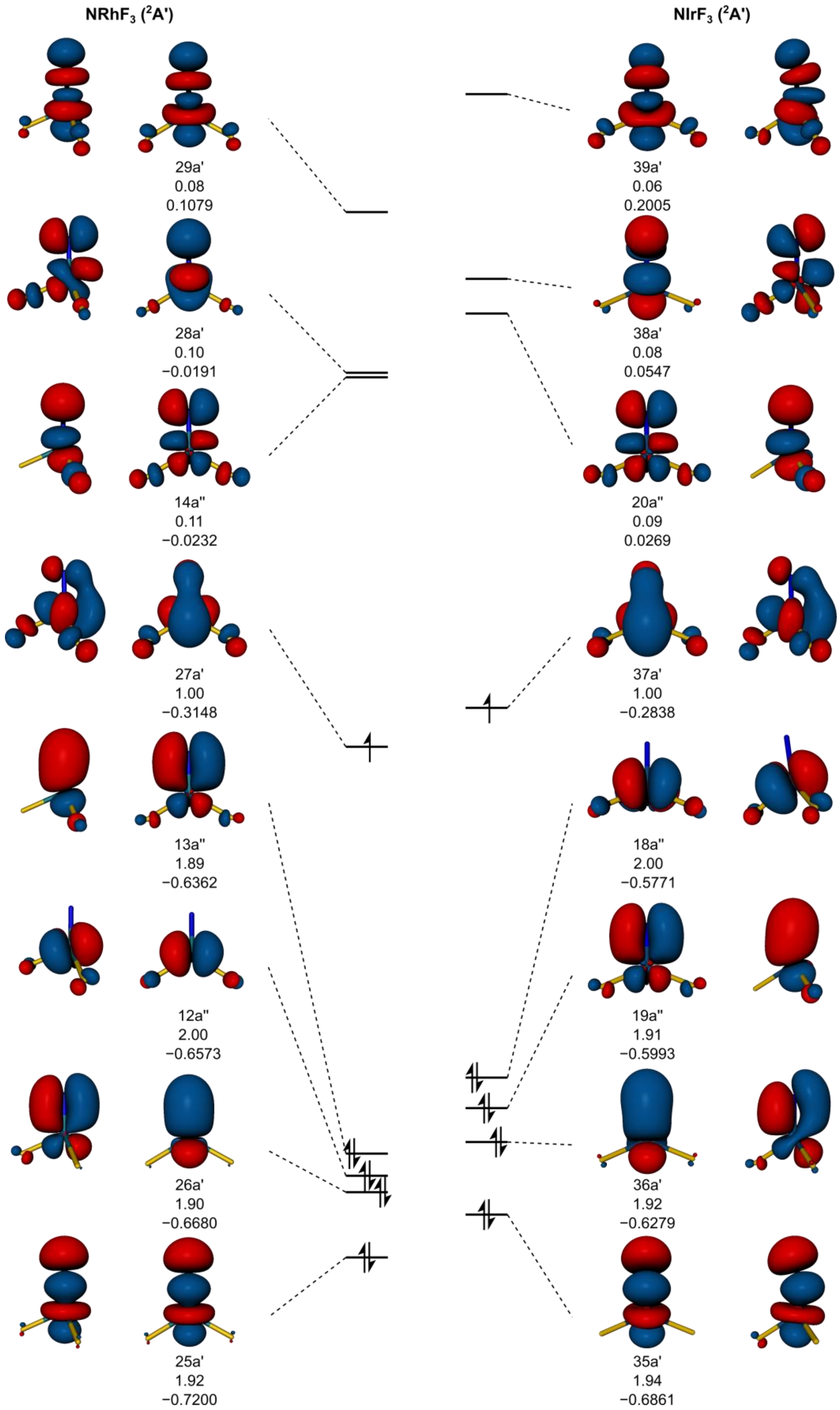

Figure S8. MO diagram of the active space natural molecular orbitals, irreproducible representations, occupation numbers and orbital energies of $NRhF_3$ (left) and $NIrF_3$ (right) calculated at the CASSCF(9,8)/cc-pVTZ-DK level of theory at the B3LYP/def2-QZVP optimized ground state structures.

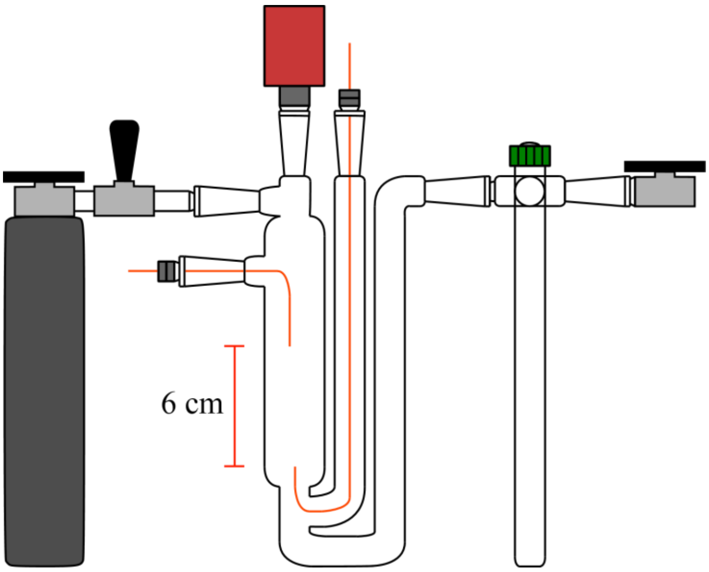

Figure S9. Electric discharge cell attached with a stainless-steel cylinder containing premixed $^{15}N_2$ and F_2 (left) and a dried glass for the final product (right).

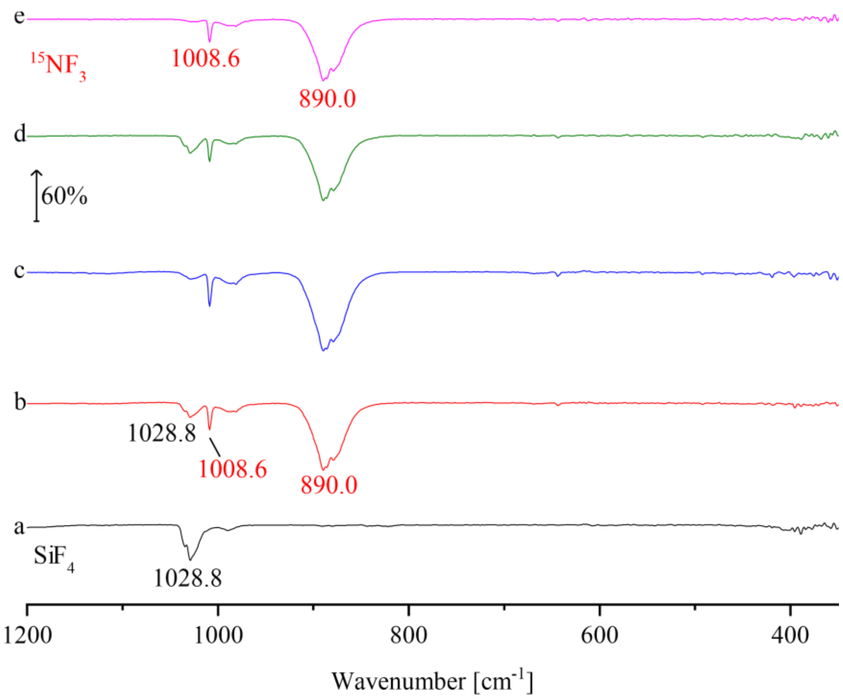

Figure S10. Gas phase IR spectra of the most volatile components of crude product during the purification process of $^{15}NF_3$ by gradually reducing the pressure to 10^{-3} mbar (e).

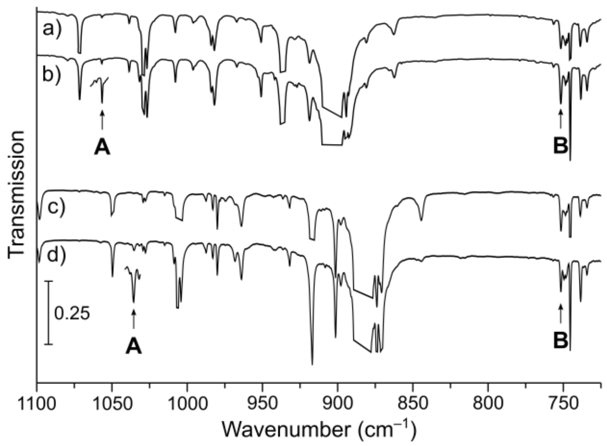

Figure S11. Infrared transmission spectra in the 1100–725 cm^{-1} region after co-depositing laser-ablated cobalt atoms with 0.1 % $^{14}NF_3$ (a), with $^{14}NF_3$ after annealing to 10 K (b) as well as with $^{15}NF_3$ (c), with subsequent annealing to 10 K (d). The absorption bands belonging to the F-N stretching mode and antisymmetric F-Co-F stretching mode are labeled **A** and **B**, respectively.

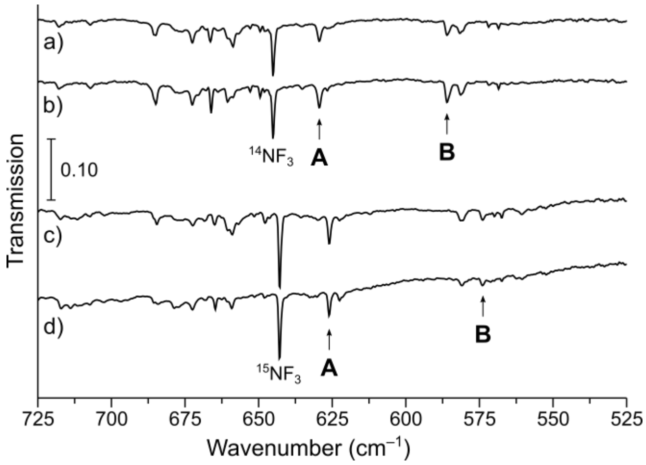

Figure S12. Infrared transmission spectra in the 725–525 cm^{-1} region after co-depositing laser-ablated cobalt atoms with 0.1 % $^{14}NF_3$ (a), with $^{14}NF_3$ after annealing to 10 K (b) as well as with $^{15}NF_3$ (c), with subsequent annealing to 10 K (d). The absorption bands belonging to the symmetric F-Co-F stretching mode and Co-N stretching mode are labeled **A** and **B**, respectively.

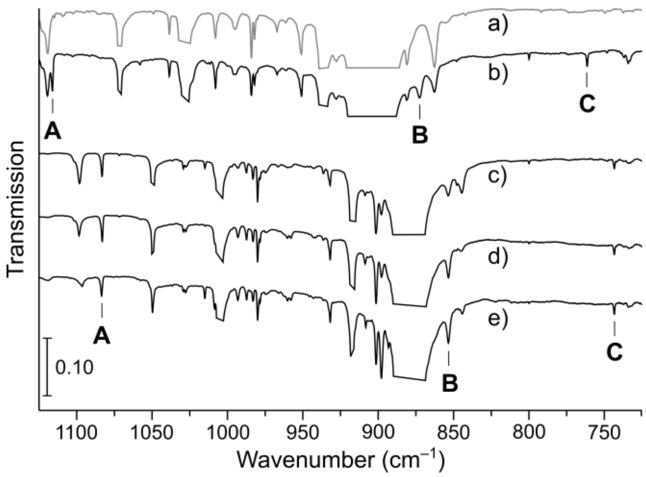

Figure S13. Infrared transmission spectra in the 1125–725 cm^{-1} region after co-depositing laser-ablated iridium atoms with 0.1 % $^{14}NF_3$ (a), co-depositing rhodium with $^{14}NF_3$ (b) as well as $^{15}NF_3$ (c), with subsequent full-arc photolysis (d) and annealing to 12 K (e). The absorption bands belonging to the N-Rh stretching mode of $NRhF_3$, Rh-N-F stretching mode and to the symmetric Rh-N-F stretching mode are labeled **A**, **B** and **C**. For the sake of cleartity, some very strong NF_3 absorption bands were cropped.

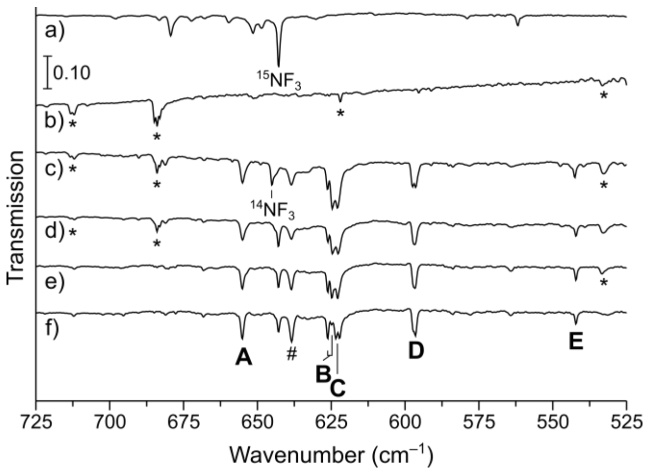

Figure S14. Infrared transmission spectra in the 725–525 cm^{-1} region after co-depositing laser-ablated iridium atoms with 0.1 % $^{15}NF_3$ (a), co-depositing rhodium with 0.1 % F_2 (b), with 0.1 % $^{14}NF_3$ (c), with 0.1 % $^{15}NF_3$ (d), after full arc photolysis (e) and annealing to 12 K (f). The absorption bands belonging to the antisymmetric and symmetric F-Rh-F stretching mode of $FNRhF_2$ are labeled with **A** and **D**. The absorption bands stemming from the antisymmetric and symmetric F-Rh-F stretching, as well as the F'-Rh stretching mode of $NRhF_3$ are labeled with **B**, **C** and **E**, respectively. Bands marked with asterisks are assigned to rhodium fluorides.

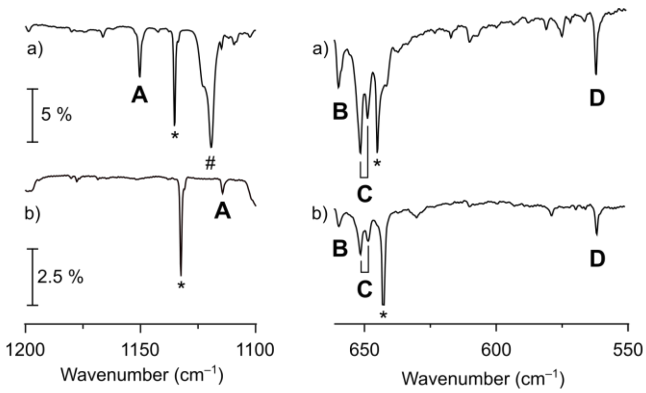

Figure S15. Infrared transmission spectra in the 1200–1100 cm^{-1} (left) and 660–550 cm^{-1} (right) region from co-deposition of laser-ablated iridium atoms with 0.1 % $^{14}NF_3$ (a) and 0.1 % $^{15}NF_3$ in neon. Bands highlighted. Labels **A**, **B, C** and **D** denote the absorption bands of $NIrF_3$. Namely, the Ir-N stretching mode, the symmetric F-Ir-F stretching mode, the antisymmetric F-Ir-F stretching mode and the Ir-F' stretching mode, respectively. Labels ***** and # denote absorptions bands from NF_3 and NF, respectively.

Supporting Tables

Table S1. Reaction enthalpies at T = 0 for reactions of formation and selected decomposition reactions obtained at the B3LYP and BP86 level of theory. All complexes in Cs point group symmetry, unless stated otherwise.

**Reaction**		**ΔH^0 [kJ mol^{-1}]**	
		**B3LYP**	**BP86**
Co + NF_3 ($^1A_1$)	→ F_2NCoF ($^4A_2$ – C_{2v})	−470	−441
F_2NCoF ($^4A_2$ – C_{2v})	→ $FNCoF_2$ (^{2}A'')	−92	−158
$FNCoF_2$ (^{2}A'')	→ $NCoF_3$ (^{2}A'')	77	47
$FNCoF_2$ (^{2}A'')	→ $NCoF_2$ ($^1A_1$ – C_{2v}) + F	331	290
$FNCoF_2$ (^{2}A'')	→ $NCoF_2$ ($^3B_1$ – C_{2v}) + F	311	315
$FNCoF_2$ (^{2}A'')	→ $NCoF_2$ ($^1A_1$ – C_{2v}) + ½ F_2	258	187
$FNCoF_2$ (^{2}A'')	→ $NCoF_2$ ($^3B_1$ – C_{2v}) + ½ F_2	238	212
$FNCoF_2$ (^{2}A'')	→ NCoF (^{4}A'') + F_2	522	533
$FNCoF_2$ (^{2}A'')	→ NCoF (^{2}A') + F_2	522	493
Rh + NF_3 ($^1A_1$)	→ F_2NRhF (^{2}A')	−356	−413
F_2NRhF (^{2}A')	→ $FNRhF_2$ (^{2}A')	−136	−155
$FNRhF_2$ (^{2}A')	→ $NRhF_3$ (^{2}A') [a)]	−7	−26
$NRhF_3$ (^{2}A')	→ $NRhF_2$ ($^1A_1$ – C_{2v}) + F	168	221
$NRhF_3$ (^{2}A')	→ $NRhF_2$ ($^1A_1$ – C_{2v}) + ½ F	95	118
$NRhF_3$ (^{2}A')	→ NRhF (^{2}A') + F_2	386	423
Ir + NF_3 ($^1A_1$)	→ F_2NIrF (^{2}A')	−460	−509
F_2NIrF (^{2}A')	→ $FNIrF_2$ (^{2}A'')	−186	−201
$FNIrF_2$ (^{2}A'')	→ $NIrF_3$ (^{2}A') [b)]	−81	−77
$NIrF_3$ (^{2}A')	→ $NIrF_2$ ($^1A_1$) + F	197	240
$NIrF_3$ (^{2}A')	→ $NIrF_2$ ($^1A_1$) + ½ F	248	274
$NIrF_3$ (^{2}A')	→ NIrF (^{2}A') + F_2	444	468

[a)]: CCSD(T) at B3LYP minimum: ΔH^0 = −12 kJ mol^{-1}; [b)]: CCSD(T) at B3LYP minimum: ΔH^0 = −98 kJ mol^{-1}

Table S2. Assignments of IR absorptions of 0.1 % $^{15}NF_3$ isolated in solid neon (Figure S2).

Assignment	Frequency [cm^{-1}]	Intensity*	Isotopic Shift [cm^{-1}]	Lit.[14]
$\nu_1 + \nu_3$	1879.1	m		-
$2\nu_3$	1756.7	m		-
$\nu_2 + \nu_3$	1517.5	w		-
$\nu_1 + \nu_4$	1496.3	w		-
$2\nu_2$	1277.4	m		-
$\nu_2 + \nu_4$	1132.3	m		-
ν_1	1006.2	s	–22.9	1008.9
$2\nu_4$	979.7	m		981.9
ν_3	879.9	vs	–21.5	886.3
ν_2	642.7	m		644.8
ν_4	491.6	m		492.0

Table S3. Selected structural parameters calculated at various levels of theory for $FNMNF_2$ (M = Co, Rh) species. Bond lengths in pm, angles in degrees.

	r(FN)		r(NM)		r(MF)		a(FNM)		a(FMF)	
**Method**	**$FNCoF_2$**	**$FNRhF_2$**	**$FNCoF_2$**	**$FNRhF_2$**	**$FNCoF_2$**	**$FNRhF_2$**	**$FNCoF_2$**	**$FNRhF_2$**	**$FNCoF_2$**	**$FNRhF_2$**
CASPT2	131	135	177	174	171	186	113	113	148	135
B3LYP	132	137	183	170	173	188	113	115	148	131
BP86 a)	138	142	162	172	172	186/191	115	117	133	131

[a]: BP86 converged to a structure with C_1 point group symmetry for $FNRhF_2$.

Table S4. Weights of the main configurations of the electronic ground states of $FNCoF_2$ ($^2A''$, Cs) and $FNRhF_2$ ($^2A'$, C_s) obtained at the CASSCF(9,7)/cc-pVTZ-DK level of theory.

Configuration a)	Weight [%]
**$FNCoF_2$ ($^2A''$, C_s)**	
$\sigma^2\ \pi^2\ \delta^1\ \pi^{*0}\ \sigma^{*0}$	48
$\sigma^2\ \pi^0\ \delta^1\ \pi^{*2}\ \sigma^{*0}$	10
$\sigma^2\ \pi^1\ \delta^1\ \pi^{*1}\ \sigma^{*2}$	6
$\sigma^0\ \pi^2\ \delta^1\ \pi^{*0}\ \sigma^{*2}$	5
$\sigma^0\ \pi^0\ \delta^1\ \pi^{*2}\ \sigma^{*2}$	4
**$FNRhF_2$ ($^2A'$, C_s)**	
$\sigma^2\ \pi^2\ \delta^1\ \pi^{*0}\ \sigma^{*0}$	81
$\sigma^2\ \pi^0\ \delta^1\ \pi^{*2}\ \sigma^{*0}$	5

[a] Approximate local symmetries are used for an ostensive description the frontier molecular orbitals along the M-N bond axis. σ, π, δ, σ*, π* and δ* correspond to 21a', 9a'', 10a'', 11a'' and 22a' ($FNCoF_2$) and 26a', 13a'', 27a', 14a'' and 28a' ($FNRhF_2$) (see Figure S6).

Table S5. Comparison of infrared band positions (cm^{-1}) and isotopic shifts (cm^{-1}, in parenthesis) of $FNRhF_2$ ($^2A'$ - C_s) observed in solid neon with values calculated using CCSD(T) with different basis set qualities, and CASPT2.

Assignment	Neon	cc-pVDZ(-PP)[a)]	aug-cc-pVTZ(-PP)[a)]	cc-pVTZ-DK[b)]	CASPT2
F-N str. [a']	872.6 (−18.9)	893 (−16)	872 (−16)	899 (−17)	981 (−19)
N-Rh str. [a']	761.4 (−18.0)	536 (−15)	707 (−19)	720 (−18)	756 (−20)
antisym. F-Rh-F str. [a'']	655.1 (0)	665 (0)	652 (0)	663 (0)	672 (0)
sym. F-Rh-F str. [a']	596.7 (0)	620 (−2)	580 (−2)	603 (−2)	623 (−0.5)

[a]: RHF/UCCSD(T) using ECP28MDF. [b]: RHF/UCCSD(T) using DKH2 relativistic approximation.

Detailed Computational Results

All structures in C_s point group symmetry (unless stated otherwise) and with positive HOMO-LUMO or SOMO-LUMO gap.

$NCoF_3$

$NCoF_3$ ($^2A''$)

UB3LYP/def2-QZVP

Cartesian coordinates

```
5
Energy = -1736.977510164
Co   -0.0824265    0.1971398    0.0000000
N     0.0349649   -1.6577570    0.0000000
F    -0.8083289    0.4847773   -1.5498606
F    -0.8083289    0.4847773    1.5498606
F     1.6641194    0.4910626    0.0000000
```

Vibrational data (^{14}N)

```
#  mode     symmetry     wave number   IR intensity    selection rules
#                         cm**(-1)        km/mol         IR     RAMAN
     7        a"             59.35        30.72704       YES     YES
     8        a'            105.26         1.24646       YES     YES
     9        a"            167.56         1.25348       YES     YES
    10        a'            172.03        11.22983       YES     YES
    11        a'            178.97        23.98249       YES     YES
    12        a'            350.87        14.14127       YES     YES
    13        a'            641.89        16.50932       YES     YES
    14        a'            669.44       116.12893       YES     YES
    15        a"            681.27       219.92593       YES     YES

zero point VIBRATIONAL energy  :      0.0068952  Hartree
```

UBP86/def2-QZVP

Cartesian coordinates

```
5
Energy = -1737.485783464
Co    0.0728443   -0.1192190    0.0000000
N    -0.0208896   -1.6287334    0.0000000
F    -0.8502485    0.5715962   -1.3132437
F    -0.8502485    0.5715962    1.3132437
F     1.6485423    0.6047601    0.0000000
```

Vibrational data (^{14}N)

```
#  mode     symmetry     wave number   IR intensity    selection rules
#                         cm**(-1)        km/mol         IR     RAMAN
     1        a"            -84.40         0.00000       YES     YES
     2                       -0.00         0.00000        -       -
     3                       -0.00         0.00000        -       -
     4                        0.00         0.00000        -       -
     5                        0.00         0.00000        -       -
     6                        0.00         0.00000        -       -
     7                        0.00         0.00000        -       -
     8        a'            155.54         2.71508       YES     YES
     9        a"            194.10         2.25560       YES     YES
    10        a'            232.19         5.74052       YES     YES
    11        a'            305.27         0.33614       YES     YES
    12        a'            632.77        35.06050       YES     YES
    13        a"            633.32        70.40555       YES     YES
    14        a'            673.32        82.23947       YES     YES
    15        a'           1064.23        38.95532       YES     YES

zero point VIBRATIONAL energy  :      0.0088637  Hartree
```

$NCoF_3$ ($^2A'$)

UB3LYP/def2-QZVP

Cartesian coordinates

```
5
Energy = -1736.964585276
Co   -0.1036774    0.0986648    0.0000000
N    -0.0159914   -1.6090243    0.0000000
F    -0.7092819    0.4651010   -1.5717060
F    -0.7092819    0.4651010    1.5717060
F     1.5382325    0.5801575    0.0000000
```

Vibrational data (^{14}N)

```
#  mode     symmetry     wave number   IR intensity    selection rules
#                         cm**(-1)        km/mol         IR     RAMAN
      7        a"            136.23         3.27842       YES     YES
      8        a'            146.67         7.70575       YES     YES
      9        a'            186.57        15.49275       YES     YES
     10        a"            202.06         0.30524       YES     YES
     11        a'            214.74         7.02491       YES     YES
     12        a'            445.19         7.28441       YES     YES
     13        a'            648.89        25.69070       YES     YES
     14        a'            711.12        78.98532       YES     YES
     15        a"            730.09       154.14645       YES     YES

zero point VIBRATIONAL energy  :      0.0077949  Hartree
```

UBP86/def2-QZVP

Cartesian coordinates

```
5
Energy = -1737.485954003
Co   -0.0826662   -0.1198141    0.0000000
N     0.0413591   -1.6272030    0.0000000
F    -0.7391524    0.5790938   -1.4494765
F    -0.7391524    0.5790938    1.4494765
F     1.5196121    0.5888295    0.0000000
```

Vibrational data (^{14}N)

```
#  mode     symmetry     wave number   IR intensity    selection rules
#                         cm**(-1)        km/mol         IR     RAMAN
      7        a"             96.35        14.57294       YES     YES
      8        a'            135.96         2.70331       YES     YES
      9        a"            208.18         1.48976       YES     YES
     10        a'            233.29         6.05019       YES     YES
     11        a'            301.59         1.36632       YES     YES
     12        a'            633.56        35.02780       YES     YES
     13        a'            643.95        55.50206       YES     YES
     14        a"            665.89        99.37128       YES     YES
     15        a'           1063.94        40.79076       YES     YES

zero point VIBRATIONAL energy  :      0.0090733  Hartree
```

Vibrational data (^{15}N)

```
#  mode     symmetry     wave number   IR intensity    selection rules
#                         cm**(-1)        km/mol         IR     RAMAN
      7        a"             96.16        14.66095       YES     YES
      8        a'            135.79         2.64381       YES     YES
      9        a"            204.32         1.40225       YES     YES
     10        a'            232.82         6.01191       YES     YES
     11        a'            296.11         1.48385       YES     YES
     12        a'            632.86        34.36728       YES     YES
     13        a'            643.94        55.51619       YES     YES
     14        a"            665.87        99.40245       YES     YES
     15        a'           1035.35        40.36567       YES     YES

zero point VIBRATIONAL energy  :      0.0089833  Hartree
```

$NCoF_3$ ($^4A'$)

UB3LYP/def2-QZVP

Cartesian coordinates

```
5
Energy = -1736.959281210
Co   -0.0580699    0.0719537    0.0000000
N     0.0662034   -1.5643483    0.0000000
F    -0.8334018    0.5109545   -1.4886131
F    -0.8334018    0.5109545    1.4886131
F     1.6586701    0.4704857    0.0000000
```

Vibrational data (^{14}N)

```
#  mode     symmetry     wave number   IR intensity    selection rules
#                         cm**(-1)        km/mol         IR     RAMAN
      7        a"            131.43        20.84005       YES     YES
      8        a'            160.01         6.70559       YES     YES
      9        a'            200.18         9.67781       YES     YES
     10        a"            206.95         4.28889       YES     YES
     11        a'            219.46         6.78440       YES     YES
     12        a'            582.80        13.89695       YES     YES
     13        a"            652.65       181.26272       YES     YES
     14        a'            655.46        24.57878       YES     YES
     15        a'            671.43        96.45802       YES     YES

zero point VIBRATIONAL energy  :      0.0079288  Hartree
```

UBP86/def2-QZVP

Cartesian coordinates

```
5
Energy = -1737.472854233
Co   -0.0434505    0.0123310    0.0000000
N     0.1221596   -1.5603138    0.0000000
F    -0.8764964    0.5539332   -1.4272844
F    -0.8764964    0.5539332    1.4272844
F     1.6742837    0.4401164    0.0000000
```

Vibrational data (^{14}N)

```
#  mode     symmetry     wave number   IR intensity    selection rules
#                         cm**(-1)        km/mol         IR     RAMAN
      7        a"            118.94         1.18366       YES     YES
      8        a'            162.37         4.68961       YES     YES
      9        a'            208.98         5.47374       YES     YES
     10        a"            237.30         9.10538       YES     YES
     11        a'            257.04         1.46665       YES     YES
     12        a'            609.22        23.16261       YES     YES
     13        a"            619.81       110.15532       YES     YES
     14        a'            644.64        73.47093       YES     YES
     15        a'            943.64         2.85718       YES     YES

zero point VIBRATIONAL energy  :      0.0086615  Hartree
```

$FNCoF_2$

$FNCoF_2$ ($^2A'$)

UB3LYP/def2-QZVP

Cartesian coordinates

```
5
Energy = -1737.006373197
Co    0.6679205    0.1377497    0.0000000
F     0.9992619    0.6113960    1.6590249
F     0.9992619    0.6113960   -1.6590249
N    -0.7514116   -0.9950999    0.0000000
F    -1.9150327   -0.3654418    0.0000000
```

Vibrational data (^{14}N)

```
#  mode     symmetry     wave number   IR intensity    selection rules
#                         cm**(-1)        km/mol         IR     RAMAN
     7        a"             73.82         0.07915       YES     YES
     8        a'            116.38        23.64476       YES     YES
     9        a'            140.74        29.53924       YES     YES
    10        a'            214.91         5.37310       YES     YES
    11        a"            233.25         0.00917       YES     YES
    12        a'            435.21        13.13023       YES     YES
    13        a'            613.69        67.59420       YES     YES
    14        a"            723.27       166.39989       YES     YES
    15        a'           1079.89       291.07245       YES     YES

zero point VIBRATIONAL energy  :      0.0082724  Hartree
```

Vibrational data (^{15}N)

```
#  mode     symmetry     wave number   IR intensity    selection rules
#                         cm**(-1)        km/mol         IR     RAMAN
     7        a"             73.79         0.07699       YES     YES
     8        a'            116.31        23.60588       YES     YES
     9        a'            140.71        29.54361       YES     YES
    10        a'            213.80         5.30993       YES     YES
    11        a"            227.47         0.01604       YES     YES
    12        a'            422.53        12.00386       YES     YES
    13        a'            613.49        68.33462       YES     YES
    14        a"            723.26       166.40925       YES     YES
    15        a'           1059.92       277.78702       YES     YES

zero point VIBRATIONAL energy  :      0.0081815  Hartree
```

UBP86/def2-QZVP

Cartesian coordinates

```
5
Energy = -1737.500231984
Co    0.5467649    0.0561976    0.0000000
F     1.0761863    0.6485766    1.5474194
F     1.0761863    0.6485766   -1.5474194
N    -0.7185615   -0.9438777    0.0000000
F    -1.9805759   -0.4094731    0.0000000
```

Vibrational data (^{14}N)

```
#  mode     symmetry     wave number   IR intensity    selection rules
#                         cm**(-1)        km/mol         IR     RAMAN
     7        a"            112.51         0.43116       YES     YES
     8        a'            138.65         7.71356       YES     YES
     9        a'            166.08        17.58136       YES     YES
    10        a'            276.60         1.94540       YES     YES
    11        a"            351.05         1.14144       YES     YES
    12        a'            616.31        36.43540       YES     YES
    13        a"            722.19       100.77692       YES     YES
    14        a'            741.09       122.74231       YES     YES
    15        a'            932.95       170.49928       YES     YES

zero point VIBRATIONAL energy  :      0.0092435  Hartree
```

Vibrational data (^{15}N)

```
#  mode     symmetry     wave number   IR intensity    selection rules
#                         cm**(-1)        km/mol         IR     RAMAN
     7        a"            112.49         0.42856       YES     YES
     8        a'            138.37         7.69169       YES     YES
     9        a'            166.04        17.55108       YES     YES
    10        a'            274.06         1.94428       YES     YES
    11        a"            342.20         1.00243       YES     YES
    12        a'            613.18        32.56996       YES     YES
    13        a"            722.09       100.89330       YES     YES
    14        a'            729.61       121.56463       YES     YES
    15        a'            911.38       165.26980       YES     YES

zero point VIBRATIONAL energy  :      0.0091341  Hartree
```

CASPT2/cc-pVTZ-DK

Cartesian coordinates

```
    5
 RS2C/USERDEF  ENERGY=-1746.62362045
 N         -0.5955943213       -1.2875004164       -0.0000000000
 F          0.3679036402       -2.1752735487       -0.0000000000
 Co        -0.0886500391        0.3767507245       -0.0000000000
 F          0.1730977030        0.9779040322        1.6014094830
 F          0.1730977030        0.9779040322       -1.6014094830
```

Vibrational data (^{14}N)

```
Vibration        Wavenumber
   Nr              [1/cm]
   1                76.66
   2               140.92
   3               169.96
   4               235.12
   5               286.35
   6               636.09
   7               673.16
   8               756.86
   9              1165.69

Zero point energy:  0.00943348 [H]     2070.41 [1/CM]       24.77 [KJ/MOL]
```

Vibrational data (^{15}N)	Vibration Wavenumber<br>Nr [1/cm]<br>1 76.52<br>2 140.76<br>3 169.89<br>4 229.59<br>5 284.29<br>6 622.31<br>7 670.29<br>8 756.86<br>9 1142.42<br><br>Zero point energy: 0.00932434 [H] 2046.46 [1/CM] 24.48 [KJ/MOL]
**$FNCoF_2$ ($^2A''$)**	
UB3LYP/def2-QZVP	
Cartesian coordinates	5<br>Energy = -1737.008529542<br>Co 0.6578907 0.2282534 0.0000000<br>F 0.9933517 0.5639077 1.6648322<br>F 0.9933517 0.5639077 -1.6648322<br>N -0.7297857 -0.9699712 0.0000000<br>F -1.9148085 -0.3860975 0.0000000
Vibrational data (^{14}N)	# mode symmetry wave number IR intensity selection rules<br># cm**(-1) km/mol IR RAMAN<br>7 a" 86.22 0.00738 YES YES<br>8 a' 136.01 21.15162 YES YES<br>9 a' 156.35 16.81925 YES YES<br>10 a' 229.94 6.05818 YES YES<br>11 a" 242.76 0.04905 YES YES<br>12 a' 441.58 11.52011 YES YES<br>13 a' 610.87 60.15444 YES YES<br>14 a" 763.84 170.91208 YES YES<br>15 a' 1079.65 293.27012 YES YES<br><br>zero point VIBRATIONAL energy : 0.0085368 Hartree
Vibrational data (^{15}N)	# mode symmetry wave number IR intensity selection rules<br># cm**(-1) km/mol IR RAMAN<br>7 a" 86.19 0.00688 YES YES<br>8 a' 135.87 21.13980 YES YES<br>9 a' 156.31 16.78193 YES YES<br>10 a' 228.82 6.05007 YES YES<br>11 a" 236.72 0.05756 YES YES<br>12 a' 428.68 10.54890 YES YES<br>13 a' 610.80 60.60073 YES YES<br>14 a" 763.84 170.91249 YES YES<br>15 a' 1059.62 279.81559 YES YES<br><br>zero point VIBRATIONAL energy : 0.0084448 Hartree
UBP86/def2-QZVP	
Cartesian coordinates	5<br>Energy = -1737.504066547<br>Co 0.5575227 0.1057569 0.0000000<br>F 1.0263887 0.6095572 1.5790247<br>F 1.0263887 0.6095572 -1.5790247<br>N -0.6776175 -0.9507779 0.0000000<br>F -1.9326826 -0.3740934 0.0000000
Vibrational data (^{14}N)	# mode symmetry wave number IR intensity selection rules<br># cm**(-1) km/mol IR RAMAN<br>7 a" 98.33 0.01475 YES YES<br>8 a' 145.38 10.86359 YES YES<br>9 a' 155.70 7.50066 YES YES<br>10 a" 293.08 0.59630 YES YES<br>11 a' 309.92 1.36285 YES YES<br>12 a' 626.80 62.87264 YES YES<br>13 a" 731.64 122.80177 YES YES<br>14 a' 764.99 36.78948 YES YES<br>15 a' 869.57 220.12138 YES YES<br><br>zero point VIBRATIONAL energy : 0.0091022 Hartree
Vibrational data (^{15}N)	# mode symmetry wave number IR intensity selection rules<br># cm**(-1) km/mol IR RAMAN<br>7 a" 98.19 0.01322 YES YES<br>8 a' 145.37 10.83214 YES YES<br>9 a' 155.53 7.52429 YES YES<br>10 a" 286.02 0.60140 YES YES<br>11 a' 307.58 1.30870 YES YES<br>12 a' 625.63 61.79012 YES YES<br>13 a" 731.64 122.80493 YES YES<br>14 a' 747.66 33.35537 YES YES<br>15 a' 850.86 214.20949 YES YES<br><br>zero point VIBRATIONAL energy : 0.0089953 Hartree
**CASPT2/cc-pVTZ-DK**	
Cartesian coordinates	5<br>RS2C/USERDEF ENERGY=-1746.62921561<br>N -0.6242290885 -1.2490400345 -0.0000000000<br>F 0.3476754071 -2.1288914983 -0.0000000000<br>Co -0.0513718917 0.4289961249 -0.0000000000<br>F 0.1359488491 0.8595025921 1.6401541026<br>F 0.1359488491 0.8595025921 -1.6401541026
Vibrational data (^{14}N)	Vibration Wavenumber<br>Nr [1/cm]<br>1 131.49<br>2 157.58

```
   3             182.22
   4             298.34
   5             393.72
   6             605.28
   7             650.95
   8             859.03
   9            1151.77

Zero point energy:  0.01009317 [H]     2215.19 [1/CM]       26.50 [KJ/MOL]
```

Vibrational data (^{15}N)

```
Vibration        Wavenumber
   Nr              [1/cm]
   1             131.48
   2             157.43
   3             182.15
   4             296.62
   5             383.87
   6             589.22
   7             650.15
   8             858.73
   9            1129.33

Zero point energy:  0.00997606 [H]     2189.49 [1/CM]       26.19 [KJ/MOL]
```

$FNCoF_2$ ($^4A''$)

UB3LYP/def2-QZVP

Cartesian Coordinates

```
5
Energy = -1736.990866197
Co    0.5683084    0.2635693    0.0000000
F     1.3053751    0.6112250    1.5383805
F     1.3053751    0.6112250   -1.5383805
N    -0.9991972   -0.4730433    0.0000000
F    -2.1798615   -1.0129759    0.0000000
```

Vibrational data (^{14}N)

```
#  mode     symmetry     wave number   IR intensity    selection rules
#                         cm**(-1)        km/mol          IR     RAMAN
     1        a"           -117.18         0.00000       YES     YES
     2                       -0.00         0.00000        -       -
     3                       -0.00         0.00000        -       -
     4                        0.00         0.00000        -       -
     5                        0.00         0.00000        -       -
     6                        0.00         0.00000        -       -
     7                        0.00         0.00000        -       -
     8        a'             55.22         2.36818       YES     YES
     9        a'            153.03        10.11935       YES     YES
    10        a"            192.11         4.02763       YES     YES
    11        a'            203.07        22.47860       YES     YES
    12        a'            409.09         1.29660       YES     YES
    13        a'            657.68       102.21115       YES     YES
    14        a"            712.90       154.54927       YES     YES
    15        a'           1265.61       378.12091       YES     YES
```

UBP86/def2-QZVP

Cartesian coordinates

```
5
Energy = -1737.478861591
Co    0.5189827    0.2408345    0.0000000
F     1.3212532    0.6175159    1.4943089
F     1.3212532    0.6175159   -1.4943089
N    -0.9813842   -0.4592354    0.0000000
F    -2.1801049   -1.0166309    0.0000000
```

Vibrational data (^{14}N)

```
#  mode     symmetry     wave number   IR intensity    selection rules
#                         cm**(-1)        km/mol          IR     RAMAN
     1        a"           -100.01         0.00000       YES     YES
     2                       -0.00         0.00000        -       -
     3                       -0.00         0.00000        -       -
     4                       -0.00         0.00000        -       -
     5                       -0.00         0.00000        -       -
     6                       -0.00         0.00000        -       -
     7                       -0.00         0.00000        -       -
     8        a'            107.66         2.89045       YES     YES
     9        a'            165.12         3.70206       YES     YES
    10        a'            187.24        12.16159       YES     YES
    11        a"            249.71         2.68147       YES     YES
    12        a'            504.88         1.45047       YES     YES
    13        a'            658.70       113.48983       YES     YES
    14        a"            699.55        99.92836       YES     YES
    15        a'           1203.90       335.32383       YES     YES

zero point VIBRATIONAL energy  :      0.0086041  Hartree
```

F_2NCoF

F_2NCoF ($^2A'$)

UB3LYP/def2-QZVP

Cartesian coordinates

```
5
Energy = -1736.956358175
N     0.5926403    0.4164280    0.0000000
F     1.4009083    0.6604467    1.0728089
F     1.4009083    0.6604467   -1.0728089
Co   -1.0109650   -0.3199323    0.0000000
F    -2.3834919   -1.4173891    0.0000000
```

Vibrational data (^{14}N)

```
#  mode     symmetry     wave number   IR intensity    selection rules
#                         cm**(-1)        km/mol          IR     RAMAN
     7        a'             57.91        12.09354       YES     YES
     8        a"            106.98        11.78200       YES     YES
     9        a"            236.73         7.17646       YES     YES
    10        a'            270.78         3.10322       YES     YES
```

	11 a' 332.51 1.31858 YES YES<br>12 a' 572.20 1.74459 YES YES<br>13 a' 680.93 160.22754 YES YES<br>14 a" 897.33 158.22600 YES YES<br>15 a' 1067.13 324.70384 YES YES<br><br>zero point VIBRATIONAL energy : 0.0096196 Hartree
UBP86/def2-QZVP	
Cartesian coordinates	5<br>Energy = -1737.441345404<br>N 0.5433949 0.3888218 0.0000000<br>F 1.3880715 0.6644346 1.0837875<br>F 1.3880715 0.6644346 -1.0837875<br>Co -0.9582474 -0.3449221 0.0000000<br>F -2.3612905 -1.3727688 0.0000000
Vibrational data (^{14}N)	# mode symmetry wave number IR intensity selection rules<br># cm**(-1) km/mol IR RAMAN<br>7 a' 38.63 7.48164 YES YES<br>8 a" 107.45 7.64337 YES YES<br>9 a" 266.90 3.49066 YES YES<br>10 a' 356.08 1.89152 YES YES<br>11 a' 411.32 0.09485 YES YES<br>12 a' 554.74 1.27295 YES YES<br>13 a" 643.20 183.28885 YES YES<br>14 a' 696.29 171.80163 YES YES<br>15 a' 925.22 249.92458 YES YES<br><br>zero point VIBRATIONAL energy : 0.0091123 Hartree
**F_2NCoF ($^4B_2$ – C_{2v})**	
UBP86/def2-QZVP	
Cartesian coordinates	5<br>Energy = -1737.443567762<br>N 0.0000000 0.0000000 0.7169257<br>F -1.0945571 0.0000000 1.5788056<br>F 1.0945571 0.0000000 1.5788056<br>Co 0.0000000 0.0000000 -1.0562770<br>F 0.0000000 0.0000000 -2.8182599
Vibrational data (^{14}N)	# mode symmetry wave number IR intensity selection rules<br># cm**(-1) km/mol IR RAMAN<br>7 b2 9.39 0.07651 YES YES<br>8 b1 85.90 13.31764 YES YES<br>9 b2 95.98 21.88961 YES YES<br>10 b1 207.10 8.88776 YES YES<br>11 a1 339.54 1.25675 YES YES<br>12 a1 554.21 0.72027 YES YES<br>13 a1 674.19 168.59633 YES YES<br>14 b1 789.70 145.89988 YES YES<br>15 a1 1031.08 200.60030 YES YES<br><br>zero point VIBRATIONAL energy : 0.0086276 Hartree
Vibrational data (^{15}N)	# mode symmetry wave number IR intensity selection rules<br># cm**(-1) km/mol IR RAMAN<br>7 b2 9.16 0.07616 YES YES<br>8 b1 85.84 13.27865 YES YES<br>9 b2 95.65 21.71159 YES YES<br>10 b1 205.38 8.74926 YES YES<br>11 a1 339.16 1.20669 YES YES<br>12 a1 551.87 0.95444 YES YES<br>13 a1 674.19 168.68674 YES YES<br>14 b1 774.20 141.39183 YES YES<br>15 a1 1005.54 190.19853 YES YES<br><br>zero point VIBRATIONAL energy : 0.0085226 Hartree
UB3LYP/def2-QZVP	
Cartesian coordinates	5<br>Energy = -1736.974544782<br>N 0.0000000 0.0000000 0.7359383<br>F -1.0853741 0.0000000 1.5786927<br>F 1.0853741 0.0000000 1.5786927<br>Co 0.0000000 0.0000000 -1.0621840<br>F 0.0000000 0.0000000 -2.8311397
Vibrational data (^{14}N)	# mode symmetry wave number IR intensity selection rules<br># cm**(-1) km/mol IR RAMAN<br>7 b2 72.71 13.05138 YES YES<br>8 b1 91.72 14.98879 YES YES<br>9 b2 152.08 18.35703 YES YES<br>10 b1 214.09 13.11832 YES YES<br>11 a1 338.79 1.63549 YES YES<br>12 a1 572.29 0.25407 YES YES<br>13 a1 675.97 196.91217 YES YES<br>14 b1 919.99 133.07714 YES YES<br>15 a1 1125.56 165.37073 YES YES<br><br>zero point VIBRATIONAL energy : 0.0094844

$NCoF_2$

**$NCoF_2$ ($^1A_1$ – C_{2v})**	
UBP86/def2-QZVP	
Cartesian coordinates	4<br>Energy = -1637.617920216

	Co 0.0000000 0.0000000 -0.0620052<br>N 0.0000000 0.0000000 -1.5539271<br>F -1.4841339 0.0000000 0.8079661<br>F 1.4841339 0.0000000 0.8079661
Vibrational data (^{14}N)	# mode symmetry wave number IR intensity selection rules<br># cm**(-1) km/mol IR RAMAN<br>7 a1 183.16 4.87774 YES YES<br>8 b2 234.94 9.57772 YES YES<br>9 b1 254.82 3.08114 YES YES<br>10 a1 657.26 39.09898 YES YES<br>11 b1 713.48 114.63394 YES YES<br>12 a1 1128.26 55.50316 YES YES<br><br>zero point VIBRATIONAL energy : 0.0072262 Hartree
Vibrational data (^{15}N)	# mode symmetry wave number IR intensity selection rules<br># cm**(-1) km/mol IR RAMAN<br>1 0.00 0.00000 - -<br>2 0.00 0.00000 - -<br>3 0.00 0.00000 - -<br>4 0.00 0.00000 - -<br>5 0.00 0.00000 - -<br>6 0.00 0.00000 - -<br>7 a1 182.90 4.85836 YES YES<br>8 b2 232.90 9.71167 YES YES<br>9 b1 249.53 3.06102 YES YES<br>10 a1 656.46 38.36220 YES YES<br>11 b1 713.48 114.64223 YES YES<br>12 a1 1098.04 54.55861 YES YES<br><br>zero point VIBRATIONAL energy : 0.0071382 Hartree
UB3LYP/def2-QZVP	
Cartesian coordinates	4<br>Energy = -1637.139673305<br>Co 0.0000000 0.0000000 -0.0616639<br>N 0.0000000 0.0000000 -1.5272705<br>F -1.4889224 0.0000000 0.7944672<br>F 1.4889224 0.0000000 0.7944672
Vibrational data (^{14}N)	# mode symmetry wave number IR intensity selection rules<br># cm**(-1) km/mol IR RAMAN<br>1 0.00 0.00000 - -<br>2 0.00 0.00000 - -<br>3 0.00 0.00000 - -<br>4 0.00 0.00000 - -<br>5 0.00 0.00000 - -<br>6 0.00 0.00000 - -<br>7 a1 181.39 8.12707 YES YES<br>8 b2 232.95 16.51720 YES YES<br>9 b1 264.48 3.08692 YES YES<br>10 a1 668.86 54.81777 YES YES<br>11 b1 727.50 133.84664 YES YES<br>12 a1 1179.98 70.54244 YES YES<br><br>zero point VIBRATIONAL energy : 0.0074158 Hartree
**$NCoF_2$ ($^3B_1$ – C_{2v})**	
UBP86/def2-QZVP	
Cartesian coordinates	4<br>Energy = -1637.569854742<br>Co 0.0000000 0.0000000 0.2451546<br>N 0.0000000 0.0000000 -1.4260783<br>F -1.7047621 0.0000000 0.5904619<br>F 1.7047621 0.0000000 0.5904619
Vibrational data (^{14}N)	# mode symmetry wave number IR intensity selection rules<br># cm**(-1) km/mol IR RAMAN<br>1 0.00 0.00000 - -<br>2 0.00 0.00000 - -<br>3 0.00 0.00000 - -<br>4 0.00 0.00000 - -<br>5 0.00 0.00000 - -<br>6 0.00 0.00000 - -<br>7 b2 78.71 33.70119 YES YES<br>8 b1 165.97 1.41811 YES YES<br>9 a1 215.60 7.81754 YES YES<br>10 a1 609.15 11.31088 YES YES<br>11 b1 726.88 148.64000 YES YES<br>12 a1 752.47 0.03273 YES YES<br><br>zero point VIBRATIONAL energy : 0.0058065 Hartree
UB3LYP/def2-QZVP	
Cartesian coordinates	4<br>Energy = -1637.126142293<br>Co 0.0000000 0.0000000 0.2955723<br>N 0.0000000 0.0000000 -1.4283857<br>F -1.7157838 0.0000000 0.5664067<br>F 1.7157838 0.0000000 0.5664067
Vibrational data (^{14}N)	# mode symmetry wave number IR intensity selection rules<br># cm**(-1) km/mol IR RAMAN<br>1 -0.00 0.00000 - -<br>2 -0.00 0.00000 - -<br>3 0.00 0.00000 - -<br>4 0.00 0.00000 - -<br>5 0.00 0.00000 - -<br>6 0.00 0.00000 - -<br>7 b2 136.15 42.68995 YES YES<br>8 a1 216.95 15.36504 YES YES

	9 b1 238.75 0.00124 YES YES<br>10 a1 605.58 7.60221 YES YES<br>11 a1 630.09 9.20571 YES YES<br>12 b1 761.68 197.35444 YES YES<br><br>zero point VIBRATIONAL energy : 0.0058986 Hartree

NCoF

NCoF (^{4}A’’)	
UBP86/def2-QZVP	
Cartesian coordinates	3<br>Energy = -1537.671464455<br>N -0.5836409 1.3722074 0.0000000<br>Co -0.4340743 -0.2015633 0.0000000<br>F 1.0177152 -1.1706441 0.0000000
Vibrational data (^{14}N)	# mode symmetry wave number IR intensity selection rules<br># cm**(-1) km/mol IR RAMAN<br>7 a' 181.46 12.10371 YES YES<br>8 a' 662.42 71.72647 YES YES<br>9 a' 958.49 31.08107 YES YES
UB3LYP/def2-QZVP	
Cartesian coordinates	3<br>Energy = -1537.265567115<br>N -0.6525997 1.4077269 0.0000000<br>Co -0.3679524 -0.1641616 0.0000000<br>F 1.0205521 -1.2435654 0.0000000
Vibrational data (^{14}N)	# mode symmetry wave number IR intensity selection rules<br># cm**(-1) km/mol IR RAMAN<br>7 a' 116.84 15.25455 YES YES<br>8 a' 640.59 72.16031 YES YES<br>9 a' 810.79 75.69130 YES YES
NCoF (^{2}A’)	
UBP86/def2-QZVP	
Cartesian coordinates	3<br>Energy = -1537.685782498<br>N -0.7454242 1.3667106 0.0000000<br>Co -0.2224294 -0.0588210 0.0000000<br>F 0.9678536 -1.3078896 0.0000000
Vibrational data (^{14}N)	# mode symmetry wave number IR intensity selection rules<br># cm**(-1) km/mol IR RAMAN<br>7 a' 90.06 11.49674 YES YES<br>8 a' 676.20 71.13325 YES YES<br>9 a' 1105.40 75.13259 YES YES<br><br>zero point VIBRATIONAL energy : 0.0042639 Hartree
Vibrational data (^{15}N)	# mode symmetry wave number IR intensity selection rules<br># cm**(-1) km/mol IR RAMAN<br>7 a' 88.77 11.46668 YES YES<br>8 a' 674.58 69.55188 YES YES<br>9 a' 1076.83 74.61935 YES YES<br><br>zero point VIBRATIONAL energy : 0.0041922 Hartree
UB3LYP/def2-QZVP	
Cartesian coordinates	3<br>Energy = -1537.264970174<br>N -0.8763492 1.3887076 0.0000000<br>Co -0.0511842 0.0281166 0.0000000<br>F 0.9275334 -1.4168241 0.0000000
Vibrational data (^{14}N)	# mode symmetry wave number IR intensity selection rules<br># cm**(-1) km/mol IR RAMAN<br>7 a' 38.02 21.48325 YES YES<br>8 a' 517.29 25.44988 YES YES<br>9 a' 736.70 96.38469 YES YES

$NRhF_3$

$NRhF_3$ (^{2}A’)	
UBP86/def2-QZVP	
Cartesian coordinates	5<br>Energy = -465.1250025960<br>Rh -0.1399902 -0.2138791 0.0000000<br>N 0.0078043 -1.8047678 0.0000000<br>F -0.6911719 0.6472559 -1.5902650<br>F -0.6911719 0.6472559 1.5902650<br>F 1.5145297 0.7241294 0.0000000
Vibrational data (^{14}N)	# mode symmetry wave number IR intensity selection rules<br># cm**(-1) km/mol IR RAMAN<br>7 a' 74.38 2.82222 YES YES<br>8 a" 134.80 7.74304 YES YES<br>9 a" 190.37 2.35006 YES YES<br>10 a' 203.77 5.17666 YES YES<br>11 a' 273.94 3.15410 YES YES<br>12 a' 562.25 45.29625 YES YES<br>13 a' 600.82 48.34327 YES YES<br>14 a" 603.09 95.77682 YES YES<br>15 a' 1087.45 47.68040 YES YES<br><br>zero point VIBRATIONAL energy : 0.0084996 Hartree

Vibrational data (^{15}N)

```
#  mode     symmetry     wave number   IR intensity    selection rules
#                         cm**(-1)        km/mol         IR     RAMAN
     7        a'             74.31         2.76875       YES     YES
     8        a"            134.54         7.90728       YES     YES
     9        a"            186.46         2.18950       YES     YES
    10        a'            203.49         5.15530       YES     YES
    11        a'            268.77         3.29529       YES     YES
    12        a'            562.25        45.28897       YES     YES
    13        a'            600.65        48.02650       YES     YES
    14        a"            603.09        95.79221       YES     YES
    15        a'           1055.07        46.03235       YES     YES

zero point VIBRATIONAL energy  :      0.0084033
```

UB3LYP/def2-QZVP

Cartesian coordinates

```
5
Energy = -464.7836586566
Rh   -0.1500554   -0.2026445    0.0000000
N     0.0043903   -1.7724057    0.0000000
F    -0.6774970    0.6284580   -1.6036316
F    -0.6774970    0.6284580    1.6036316
F     1.5006592    0.7181285    0.0000000
```

Vibrational data (^{14}N)

```
#  mode     symmetry     wave number   IR intensity    selection rules
#                         cm**(-1)        km/mol         IR     RAMAN
     7        a'             70.20         4.72915       YES     YES
     8        a"            156.24         6.00424       YES     YES
     9        a"            204.81         2.24836       YES     YES
    10        a'            208.73         7.06775       YES     YES
    11        a'            285.54         3.95289       YES     YES
    12        a'            580.83        52.32699       YES     YES
    13        a'            618.29        62.99995       YES     YES
    14        a"            625.07       117.90314       YES     YES
    15        a'           1112.98        54.55496       YES     YES

zero point VIBRATIONAL energy  :      0.0087998  Hartree
```

Vibrational data (^{15}N)

```
#  mode     symmetry     wave number   IR intensity    selection rules
#                         cm**(-1)        km/mol         IR     RAMAN
     7        a'             70.13         4.65449       YES     YES
     8        a"            156.06         6.16298       YES     YES
     9        a"            200.43         2.14226       YES     YES
    10        a'            208.43         7.04629       YES     YES
    11        a'            280.16         4.14645       YES     YES
    12        a'            580.82        52.31848       YES     YES
    13        a'            618.20        62.72236       YES     YES
    14        a"            625.06       117.91473       YES     YES
    15        a'           1079.69        52.49527       YES     YES

zero point VIBRATIONAL energy  :      0.0087003  Hartree
```

RHF-UCCSD(T)/aug-cc-pVTZ(-PP)

Cartesian coordinates

```
    5
 UCCSD(T)/USERDEF  ENERGY=-463.54661547
 Rh        -0.1626075169       -0.1814607136        0.0000000000
 N          0.0004540618       -1.7626120982        0.0000000000
 F         -0.6644731875        0.6129142971       -1.6177711634
 F         -0.6644731875        0.6129142971        1.6177711634
 F          1.4910998299        0.7182385177        0.0000000000

T1 diagnostic:  0.02947583
D1 diagnostic:  0.10755742
```

Vibrational data (^{14}N)

```
 Imaginary Vibration  Wavenumber
        Nr             [1/cm]
        1              152.69
        2              110.90

     Vibration        Wavenumber
        Nr             [1/cm]
        1              145.51
        2              168.60
        3              249.35
        4              584.46
        5              621.21
        6              631.19
        7             1164.02
```

$NRhF_3$ ($^4A''$)

UBP86/def2-QZVP

Cartesian coordinates

```
5
Energy = -465.0835381244
Rh   -0.0548919    0.0013453    0.0000000
N     0.1942306   -1.6599282    0.0000000
F    -0.9892941    0.6312355   -1.5152511
F    -0.9892941    0.6312355    1.5152511
F     1.8392496    0.3961062    0.0000000
```

Vibrational data (^{14}N)

```
#  mode     symmetry     wave number   IR intensity    selection rules
#                         cm**(-1)        km/mol         IR     RAMAN
     1        a"            -42.14         0.00000       YES     YES
     2                       -0.00         0.00000        -       -
     3                       -0.00         0.00000        -       -
     4                       -0.00         0.00000        -       -
     5                        0.00         0.00000        -       -
     6                        0.00         0.00000        -       -
     7                        0.00         0.00000        -       -
     8        a'            106.89         6.01975       YES     YES
     9        a'            168.83         6.67762       YES     YES
    10        a"            195.58         8.48916       YES     YES
    11        a'            253.77         1.06099       YES     YES
```

	12 a' 540.55 47.44788 YES YES<br>13 a" 558.62 92.27919 YES YES<br>14 a' 588.48 55.34010 YES YES<br>15 a' 928.98 1.92571 YES YES<br><br>zero point VIBRATIONAL energy : 0.0076130 Hartree

FNRhF$_2$

FNRhF$_2$ (^{2}A'')	
UBP86/def2-QZVP	
Cartesian coordinates	5<br>Energy = -465.1135362013<br>F 1.2093482 -0.1828113 -1.7446750<br>Rh 0.5438307 -0.2701357 0.0000000<br>F 1.2093482 -0.1828113 1.7446750<br>N -1.1745684 -0.3187857 0.0000000<br>F -1.7879586 0.9545439 0.0000000
Vibrational data (^{14}N)	# mode symmetry wave number IR intensity selection rules<br># cm**(-1) km/mol IR RAMAN<br>7 a' 109.43 8.69355 YES YES<br>8 a" 110.74 0.60271 YES YES<br>9 a' 129.61 4.17406 YES YES<br>10 a' 317.66 1.58379 YES YES<br>11 a" 373.02 2.45020 YES YES<br>12 a' 578.57 61.58791 YES YES<br>13 a" 656.82 106.57691 YES YES<br>14 a' 763.07 235.82196 YES YES<br>15 a' 827.30 38.51876 YES YES<br><br>zero point VIBRATIONAL energy : 0.0088079 Hartree
UB3LYP/def2-QZVP	
Cartesian coordinates	5<br>Energy = -464.7768766063<br>F 1.1953393 -0.1775565 -1.7473860<br>Rh 0.5427375 -0.2711853 0.0000000<br>F 1.1953393 -0.1775565 1.7473860<br>N -1.1749425 -0.3106740 0.0000000<br>F -1.7584735 0.9369722 0.0000000
Vibrational data (^{14}N)	# mode symmetry wave number IR intensity selection rules<br># cm**(-1) km/mol IR RAMAN<br>7 a' 110.16 13.26459 YES YES<br>8 a" 110.88 0.69375 YES YES<br>9 a' 124.88 6.46848 YES YES<br>10 a' 330.89 1.27957 YES YES<br>11 a" 337.68 0.02412 YES YES<br>12 a' 587.83 78.88401 YES YES<br>13 a" 659.78 152.91911 YES YES<br>14 a' 795.82 9.95449 YES YES<br>15 a' 886.49 264.48171 YES YES<br><br>zero point VIBRATIONAL energy : 0.0089860 Hartree
CASPT2/cc-pVTZ-DK	
Cartesian coordinates	5<br>RS2C/USERDEF ENERGY=-5135.12197299<br>N -0.6219245232 -1.3512409434 -0.0000000000<br>F 0.3762961477 -2.2540436222 -0.0000000000<br>Rh -0.0422721273 0.3294831759 -0.0000000000<br>F 0.1593917812 0.8287964603 -1.7516517214<br>F 0.1593917812 0.8287964603 1.7516517214
Vibrational data (^{14}N)	Vibration Wavenumber<br>Nr [1/cm]<br>1 114.73<br>2 136.62<br>3 155.89<br>4 320.76<br>5 326.00<br>6 636.92<br>7 694.11<br>8 712.50<br>9 996.73<br><br>Zero point energy: 0.00932740 [H] 2047.13 [1/CM] 24.49 [KJ/MOL]
Vibrational data (^{154}N)	Vibration Wavenumber<br>Nr [1/cm]<br>1 114.77<br>2 136.61<br>3 155.82<br>4 312.71<br>5 323.90<br>6 636.84<br>7 673.42<br>8 712.50<br>9 977.73<br><br>Zero point energy: 0.00921362 [H] 2022.16 [1/CM] 24.19 [KJ/MOL]
RHF-UCCSD(T)/aug-cc-pVTZ-DK	
Single Point<br>B3LYP minimum	5<br>Energy = -5134.90671671<br>F 1.1953393 -0.1775565 -1.7473860<br>Rh 0.5427375 -0.2711853 0.0000000<br>F 1.1953393 -0.1775565 1.7473860

	N -1.1749425 -0.3106740 0.0000000<br>F -1.7584735 0.9369722 0.0000000<br><br>T1 diagnostic: 0.04675588<br>D1 diagnostic: 0.19468227
**$FNRhF_2$ ($^2A'$)**	
UBP86/def2-QZVP	
Cartesian coordinates	5<br>Energy = -465.1145214309<br>F 1.2825350 -0.1937175 -1.7110184<br>Rh 0.4961048 -0.2778330 0.0000000<br>F 1.2825350 -0.1937175 1.7110184<br>N -1.2055324 -0.2895846 0.0000000<br>F -1.8556423 0.9548526 0.0000000
Vibrational data (^{14}N)	1 a" -119.10 0.00000 YES YES<br>2 -0.00 0.00000 - -<br>3 -0.00 0.00000 - -<br>4 -0.00 0.00000 - -<br>5 0.00 0.00000 - -<br>6 0.00 0.00000 - -<br>7 0.00 0.00000 - -<br>8 a" 124.41 1.00971 YES YES<br>9 a' 127.16 5.75679 YES YES<br>10 a' 165.55 10.60533 YES YES<br>11 a' 266.21 1.06385 YES YES<br>12 a' 584.86 58.81893 YES YES<br>13 a" 609.97 172.40545 YES YES<br>14 a' 731.07 190.87034 YES YES<br>15 a' 858.79 94.31362 YES YES
UB3LYP/def2-QZVP	
Cartesian coordinates	5<br>Energy = -464.7812237473<br>F 1.2674668 -0.1891948 -1.7123441<br>Rh 0.4887109 -0.2713834 0.0000000<br>F 1.2674668 -0.1891948 1.7123441<br>N -1.2087954 -0.2920414 0.0000000<br>F -1.8148491 0.9418144 0.0000000<br><br>T1 diagnostic: 0.04315135<br>D1 diagnostic: 0.16672494
Vibrational data (^{14}N)	# mode symmetry wave number IR intensity selection rules<br># cm**(-1) km/mol IR RAMAN<br>7 a" 117.14 0.66171 YES YES<br>8 a' 129.09 7.48470 YES YES<br>9 a' 171.62 14.97100 YES YES<br>10 a' 281.89 1.18747 YES YES<br>11 a" 294.87 0.16733 YES YES<br>12 a' 594.11 74.92821 YES YES<br>13 a" 640.70 149.71552 YES YES<br>14 a' 785.94 117.42896 YES YES<br>15 a' 934.66 168.23779 YES YES<br><br>zero point VIBRATIONAL energy : 0.0089988 Hartree
Vibrational data (^{15}N)	# mode symmetry wave number IR intensity selection rules<br># cm**(-1) km/mol IR RAMAN<br>7 a" 117.13 0.66421 YES YES<br>8 a' 129.00 7.48213 YES YES<br>9 a' 171.60 14.95100 YES YES<br>10 a' 279.40 1.16820 YES YES<br>11 a" 287.11 0.17883 YES YES<br>12 a' 593.84 74.60447 YES YES<br>13 a" 640.70 149.71199 YES YES<br>14 a' 769.65 106.05722 YES YES<br>15 a' 910.93 169.00734 YES YES<br><br>zero point VIBRATIONAL energy : 0.0088834 Hartree
CASPT2/cc-pVTZ-DK	
Cartesian coordinates	5<br>RS2C/USERDEF ENERGY=-5135.12094042<br>N -0.6099844490 1.3826515997 0.0000000000<br>F 0.3963720782 2.2821553746 0.0000000000<br>Rh -0.0647784664 -0.2659677470 0.0000000000<br>F 0.2021089135 -0.9304505669 1.7215153928<br>F 0.2021089135 -0.9304505669 -1.7215153928
Vibrational data (^{14}N)	Vibration Wavenumber<br>Nr [1/cm]<br>1 124.60<br>2 148.57<br>3 185.64<br>4 289.99<br>5 301.71<br>6 635.04<br>7 679.56<br>8 748.29<br>9 990.67<br><br>Zero point energy: 0.00934974 [H] 2052.03 [1/CM] 24.55 [KJ/MOL]
Vibrational data (^{154}N)	Vibration Wavenumber<br>Nr [1/cm]<br>1 124.62<br>2 148.45<br>3 185.63<br>4 287.89<br>5 293.95

	6 634.26<br>7 679.56<br>8 728.37<br>9 970.94<br><br>Zero point energy: 0.00923494 [H] 2026.84 [1/CM] 24.25 [KJ/MOL]
RHF-UCCSD(T)/cc-pVTZ-DK	
Cartesian coordinates	5<br>UCCSD(T)/USERDEF ENERGY=-5134.86624986<br>F 1.2106200830 -0.1851219494 -1.7475105656<br>Rh 0.5314767484 -0.2567589344 0.0000000000<br>F 1.2106200830 -0.1851219494 1.7475105656<br>N -1.1866524395 -0.3073004114 0.0000000000<br>F -1.7660644751 0.9343032447 0.0000000000<br><br>T1 diagnostic: 0.04315135<br>D1 diagnostic: 0.16672494
Vibrational data (^{14}N)	Vibration Wavenumber<br>Nr [1/cm]<br>1 90.45<br>2 108.67<br>3 169.77<br>4 272.41<br>5 287.29<br>6 602.96<br>7 662.87<br>8 719.53<br>9 898.79<br><br>Zero point energy: 0.00868606 [H] 1906.37 [1/CM] 22.81 [KJ/MOL]
Vibrational data (^{154}N)	Vibration Wavenumber<br>Nr [1/cm]<br>1 90.45<br>2 108.62<br>3 169.80<br>4 270.40<br>5 280.16<br>6 600.69<br>7 662.87<br>8 701.23<br>9 882.21<br><br>Zero point energy: 0.00858055 [H] 1883.21 [1/CM] 22.53 [KJ/MOL]
RHF-UCCSD(T)/cc-pVDZ(-PP)	
Cartesian coordinates	5<br>UCCSD(T)/CC-PVDZ,RH=CC-PVDZ-PP ENERGY=-463.07033821<br>F 1.1836841447 -0.1842461586 -1.7928084951<br>Rh 0.5747187837 -0.2510917318 0.0000000000<br>F 1.1836841447 -0.1842461586 1.7928084951<br>N -1.1912103992 -0.3145890842 0.0000000000<br>F -1.7508766739 0.9341731330 0.0000000000<br><br>T1 diagnostic: 0.05885526<br>D1 diagnostic: 0.22377600
Vibrational data (^{14}N)	Vibration Wavenumber<br>Nr [1/cm]<br>1 109.99<br>2 134.28<br>3 173.29<br>4 260.62<br>5 272.49<br>6 536.08<br>7 619.70<br>8 665.37<br>9 893.32<br><br>Zero point energy: 0.00834982 [H] 1832.57 [1/CM] 21.92 [KJ/MOL]
Vibrational data (^{154}N)	Vibration Wavenumber<br>Nr [1/cm]<br>1 109.99<br>2 134.25<br>3 173.26<br>4 253.74<br>5 270.58<br>6 521.43<br>7 617.56<br>8 665.37<br>9 877.43<br><br>Zero point energy: 0.00825518 [H] 1811.80 [1/CM] 21.67 [KJ/MOL]
RHF-UCCSD(T)/aug-cc-pVTZ(-PP)	
Cartesian coordinates	5<br>UCCSD(T)/USERDEF ENERGY=-463.54228861<br>F 1.2115020377 -0.1841600603 -1.7527667348<br>Rh 0.5314205692 -0.2581662671 0.0000000000<br>F 1.2115020377 -0.1841600603 1.7527667348<br>N -1.1889856678 -0.3103822366 0.0000000000<br>F -1.7654389767 0.9368686242 0.0000000000<br><br>T1 diagnostic: 0.04360507<br>D1 diagnostic: 0.17099709
Vibrational data (^{14}N)	Vibration Wavenumber<br>Nr [1/cm]<br>1 78.78<br>2 95.02

	3 168.39<br>4 262.09<br>5 265.80<br>6 580.11<br>7 651.95<br>8 706.60<br>9 872.03<br><br>Zero point energy: 0.00838543 [H] 1840.39 [1/CM] 22.02 [KJ/MOL]
Vibrational data (^{154}N)	Vibration Wavenumber<br>Nr [1/cm]<br>1 78.78<br>2 94.94<br>3 168.48<br>4 258.75<br>5 260.15<br>6 578.23<br>7 651.95<br>8 688.00<br>9 855.90<br><br>Zero point energy: 0.00828155 [H] 1817.59 [1/CM] 21.74 [KJ/MOL]
RHF-UCCSD(T)/aug-cc-pVTZ-DK	
Single Point<br>B3LYP minimum	5<br>Energy = -5134.91139993<br>F 1.2674668 -0.1891948 -1.7123441<br>Rh 0.4887109 -0.2713834 0.0000000<br>F 1.2674668 -0.1891948 1.7123441<br>N -1.2087954 -0.2920414 0.0000000<br>F -1.8148491 0.9418144 0.0000000<br><br>T1 diagnostic: 0.03825013<br>D1 diagnostic: 0.14250080
**$FNRhF_2$ (A – C_1)**	
UBP86/def2-QZVP	
Cartesian coordinates	5<br>Energy = -465.1145302452<br>F 1.0202200 0.2598587 -1.8593214<br>Rh 0.5556683 -0.1178206 -0.0714266<br>F 1.5061252 0.1304750 1.5334495<br>N -1.0571361 -0.6364492 0.1021351<br>F -2.0248775 0.3639361 0.2951633
Vibrational data (^{14}N)	# mode symmetry wave number IR intensity selection rules<br># cm**(-1) km/mol IR RAMAN<br>7 a 90.02 18.12589 YES YES<br>8 a 111.53 0.86345 YES YES<br>9 a 158.16 10.01637 YES YES<br>10 a 235.15 8.54492 YES YES<br>11 a 269.03 0.93638 YES YES<br>12 a 571.54 100.83765 YES YES<br>13 a 625.06 132.41313 YES YES<br>14 a 720.69 180.95561 YES YES<br>15 a 849.70 102.69768 YES YES<br><br>zero point VIBRATIONAL energy : 0.0082717 Hartree
Vibrational data (^{15}N)	# mode symmetry wave number IR intensity selection rules<br># cm**(-1) km/mol IR RAMAN<br>7 a 89.68 18.06742 YES YES<br>8 a 111.52 0.86782 YES YES<br>9 a 158.00 9.94902 YES YES<br>10 a 229.96 7.86173 YES YES<br>11 a 266.49 0.92703 YES YES<br>12 a 571.29 102.12900 YES YES<br>13 a 624.94 132.31129 YES YES<br>14 a 708.34 167.64499 YES YES<br>15 a 825.39 103.43826 YES YES<br><br>zero point VIBRATIONAL energy : 0.0081686 Hartree

F_2NRhF

**F_2NRhF (^{2}A')**	
UBP86/def2-QZVP	
Cartesian coordinates	5<br>Energy = -465.0566951810<br>F 1.5409517 -0.4970374 -1.0826662<br>N 0.6764619 -0.2045102 0.0000000<br>F 1.5409517 -0.4970374 1.0826662<br>Rh -0.9861563 0.3283025 0.0000000<br>F -2.7722090 0.8702825 0.0000000
Vibrational data (^{14}N)	# mode symmetry wave number IR intensity selection rules<br># cm**(-1) km/mol IR RAMAN<br>7 a' 23.69 4.39584 YES YES<br>8 a" 118.66 3.41477 YES YES<br>9 a" 323.01 5.22785 YES YES<br>10 a' 372.01 4.99836 YES YES<br>11 a' 414.95 0.89419 YES YES<br>12 a' 563.25 7.48285 YES YES<br>13 a" 571.26 198.80152 YES YES<br>14 a' 634.83 196.57650 YES YES<br>15 a' 924.64 361.91707 YES YES

UB3LYPdef2-QZVP

Cartesian coordinates

```
5
Energy = -464.7299697738
F     1.5044702   -0.2919310   -1.0703948
N     0.6479047   -0.4643889    0.0000000
F     1.5044702   -0.2919310    1.0703948
Rh   -1.0891582   -0.0518959    0.0000000
F    -2.5676868    1.1001468    0.0000000
```

Vibrational data (^{14}N)

```
#  mode     symmetry     wave number   IR intensity    selection rules
#                         cm**(-1)        km/mol         IR     RAMAN
      1                      -0.00         0.00000        -       -
      2                       0.00         0.00000        -       -
      3                       0.00         0.00000        -       -
      4                       0.00         0.00000        -       -
      5                       0.00         0.00000        -       -
      6                       0.00         0.00000        -       -
      7        a'            48.51         9.15165       YES     YES
      8        a"           139.18         4.21222       YES     YES
      9        a"           281.26         2.39126       YES     YES
     10        a'           318.72         5.69616       YES     YES
     11        a'           458.11         1.45833       YES     YES
     12        a'           588.23        14.55817       YES     YES
     13        a'           634.79       146.07276       YES     YES
     14        a"           792.39       172.82747       YES     YES
     15        a'           983.32       307.94240       YES     YES

zero point VIBRATIONAL energy  :      0.0096697  Hartree
```

F_2NRhF ($^4A''$)

UBP86/def2-QZVP

Cartesian coordinates

```
5
Energy = -465.0310830718
F     1.5733523   -0.4355884   -1.0972268
N     0.7068371   -0.4270292    0.0000000
F     1.5733523   -0.4355884    1.0972268
Rh   -1.0089743    0.4034544    0.0000000
F    -2.8445674    0.8947516    0.0000000
```

Vibrational data (^{14}N)

```
#  mode     symmetry     wave number   IR intensity    selection rules
#                         cm**(-1)        km/mol         IR     RAMAN
      7        a"            61.29         6.86247       YES     YES
      8        a'            90.91         5.67979       YES     YES
      9        a"           203.17         0.88544       YES     YES
     10        a'           218.02         2.06314       YES     YES
     11        a'           383.43         6.76419       YES     YES
     12        a'           542.70         0.85805       YES     YES
     13        a'           584.75       136.32565       YES     YES
     14        a"           770.28       157.31923       YES     YES
     15        a'           945.19       292.04257       YES     YES
```

UB3LYPdef2-QZVP

Cartesian coordinates

```
5
Energy = -464.7201593610
F     1.5674191   -0.5307839   -1.0784020
N     0.8100069   -0.1566487    0.0000000
F     1.5674191   -0.5307839    1.0784020
Rh   -1.0708510    0.2893872    0.0000000
F    -2.8739941    0.9288292    0.0000000
```

Vibrational data (^{14}N)

```
#  mode     symmetry     wave number   IR intensity    selection rules
#                         cm**(-1)        km/mol         IR     RAMAN
      1                      -0.00         0.00000        -       -
      2                      -0.00         0.00000        -       -
      3                       0.00         0.00000        -       -
      4                       0.00         0.00000        -       -
      5                       0.00         0.00000        -       -
      6                       0.00         0.00000        -       -
      7        a'            70.84         9.37160       YES     YES
      8        a"            83.74        10.26102       YES     YES
      9        a'           147.11         9.84852       YES     YES
     10        a"           225.25         2.40465       YES     YES
     11        a'           318.93         2.21753       YES     YES
     12        a'           574.41        35.77246       YES     YES
     13        a'           590.97       139.32738       YES     YES
     14        a"           904.27       150.70686       YES     YES
     15        a'          1056.89       339.36366       YES     YES

zero point VIBRATIONAL energy  :      0.0090498  Hartree
```

$NRhF_2$

$NRhF_2$ ($^1A_1$ – C_{2v})

UBP86/def2-QZVP

Cartesian coordinates

```
4
Energy = -365.2652448926
Rh    0.0000000    0.0000000   -0.1613503
F    -1.5182963    0.0000000    0.9499143
F     1.5182963    0.0000000    0.9499143
N     0.0000000    0.0000000   -1.7384783
```

Vibrational data (^{14}N)

```
#  mode     symmetry     wave number   IR intensity    selection rules
#                         cm**(-1)        km/mol         IR     RAMAN
      7        a1           146.33         5.98316       YES     YES
      8        b2           214.22         7.30914       YES     YES
      9        b1           219.44         7.86756       YES     YES
     10        a1           607.55        64.43561       YES     YES
```

	11 b1 610.72 103.94588 YES YES<br>12 a1 1151.79 63.63990 YES YES<br><br>zero point VIBRATIONAL energy : 0.0067207 Hartree
UB3LYPdef2-QZVP	
Cartesian coordinates	4<br>Energy = -364.9758642536<br>Rh 0.0000000 0.0000000 -0.1711984<br>F -1.5116457 0.0000000 0.9486391<br>F 1.5116457 0.0000000 0.9486391<br>N 0.0000000 0.0000000 -1.7260796
Vibrational data (^{14}N)	# mode symmetry wave number IR intensity selection rules<br># cm**(-1) km/mol IR RAMAN<br>7 a1 147.87 8.62397 YES YES<br>8 b2 213.90 11.57977 YES YES<br>9 b1 227.09 8.90820 YES YES<br>10 a1 617.33 83.19454 YES YES<br>11 b1 618.22 118.42241 YES YES<br>12 a1 1203.78 72.08579 YES YES<br><br>zero point VIBRATIONAL energy : 0.0068987 Hartree
Vibrational data (^{15}N)	# mode symmetry wave number IR intensity selection rules<br># cm**(-1) km/mol IR RAMAN<br>7 a1 147.79 8.61047 YES YES<br>8 b2 211.03 11.99796 YES YES<br>9 b1 222.01 8.83313 YES YES<br>10 a1 617.15 82.76296 YES YES<br>11 b1 618.21 118.48273 YES YES<br>12 a1 1167.96 69.46966 YES YES<br><br>zero point VIBRATIONAL energy : 0.0068987 Hartree
$NRhF_2$ ($^3B_2$ – C_{2v})	
Cartesian coordinates	4<br>Energy = -364.9364956873<br>Rh 0.0000000 0.0000000 0.0235522<br>F -1.7210864 0.0000000 0.7969246<br>F 1.7210864 0.0000000 0.7969246<br>N 0.0000000 0.0000000 -1.6174013
Vibrational data (^{14}N)	# mode symmetry wave number IR intensity selection rules<br># cm**(-1) km/mol IR RAMAN<br>7 a1 150.64 7.73951 YES YES<br>8 b2 202.01 20.84999 YES YES<br>9 b1 239.76 0.88301 YES YES<br>10 a1 600.19 48.90509 YES YES<br>11 b1 644.82 147.22990 YES YES<br>12 a1 1003.49 11.33293 YES YES

NRhF

**NRhF (^{2}A')**	
UBP86/def2-QZVP	
Cartesian coordinates	3<br>Energy = -265.3338973032<br>N -0.7620554 1.4838931 0.0000000<br>Rh -0.2364461 -0.0344348 0.0000000<br>F 0.9985015 -1.4494583 0.0000000
Vibrational data (^{14}N)	# mode symmetry wave number IR intensity selection rules<br># cm**(-1) km/mol IR RAMAN<br>7 a' 113.83 4.42255 YES YES<br>8 a' 622.31 89.31311 YES YES<br>9 a' 1096.97 60.63485 YES YES<br><br>zero point VIBRATIONAL energy : 0.0041762 Hartree
UB3LYPdef2-QZVP	
Cartesian coordinates	3<br>Energy = -265.0930282922<br>N -0.7500161 1.4758311 0.0000000<br>Rh -0.2509060 -0.0347037 0.0000000<br>F 1.0009221 -1.4411274 0.0000000
Vibrational data (^{14}N)	# mode symmetry wave number IR intensity selection rules<br># cm**(-1) km/mol IR RAMAN<br>7 a' 126.13 7.32084 YES YES<br>8 a' 623.80 108.50390 YES YES<br>9 a' 1135.18 65.45220 YES YES<br><br>zero point VIBRATIONAL energy : 0.0042946 Hartree
Vibrational data (^{15}N)	

$NIrF_3$

**$NIrF_3$ (^{2}A')**	
UB3LYP/def2-QZVP	
Cartesian coordinates	5<br>Energy = -458.6101089774<br>Ir -0.2324947 -0.1416380 0.0000000<br>N -0.1698665 -1.7575368 0.0000000<br>F -0.4683487 0.4787989 -1.7781147<br>F -0.4683487 0.4787989 1.7781147<br>F 1.3390586 0.9415771 0.0000000

	zero point VIBRATIONAL energy : 0.0092347 Hartree
Vibrational data (^{14}N)	# mode symmetry wave number IR intensity selection rules<br># cm**(-1) km/mol IR RAMAN<br>7 a' 115.60 1.61921 YES YES<br>8 a" 187.23 13.05511 YES YES<br>9 a' 227.00 5.07407 YES YES<br>10 a" 247.11 1.37580 YES YES<br>11 a' 268.86 4.27204 YES YES<br>12 a' 580.67 39.55680 YES YES<br>13 a" 634.01 146.61325 YES YES<br>14 a' 635.39 43.32419 YES YES<br>15 a' 1157.70 23.41349 YES YES<br><br>zero point VIBRATIONAL energy : 0.0092347 Hartree
Vibrational data (^{15}N)	# mode symmetry wave number IR intensity selection rules<br># cm**(-1) km/mol IR RAMAN<br>7 a' 115.32 1.52961 YES YES<br>8 a" 185.96 13.25430 YES YES<br>9 a' 225.79 5.20238 YES YES<br>10 a" 242.70 0.98143 YES YES<br>11 a' 265.34 4.35970 YES YES<br>12 a' 580.67 39.55760 YES YES<br>13 a" 633.98 146.72396 YES YES<br>14 a' 635.38 43.29869 YES YES<br>15 a' 1121.27 22.22143 YES YES<br><br>zero point VIBRATIONAL energy : 0.0091273 Hartree
UBP86/def2-QZVP	
Cartesian coordinates	5<br>Energy = -458.9683328816<br>Ir -0.2304637 -0.1459280 0.0000000<br>N -0.1743213 -1.7784011 0.0000000<br>F -0.4696134 0.4863221 -1.7797658<br>F -0.4696134 0.4863221 1.7797658<br>F 1.3440118 0.9516849 0.0000000<br><br>zero point VIBRATIONAL energy : 0.0089030 Hartree
Vibrational data (^{14}N)	# mode symmetry wave number IR intensity selection rules<br># cm**(-1) km/mol IR RAMAN<br>7 a' 113.14 1.09749 YES YES<br>8 a" 153.14 17.48524 YES YES<br>9 a' 223.02 3.49021 YES YES<br>10 a" 239.18 1.28440 YES YES<br>11 a' 260.10 2.87107 YES YES<br>12 a' 562.40 36.37451 YES YES<br>13 a" 617.91 131.95565 YES YES<br>14 a' 618.39 34.26185 YES YES<br>15 a' 1120.69 22.44945 YES YES<br><br>zero point VIBRATIONAL energy : 0.0089030 Hartree
Vibrational data (^{15}N)	# mode symmetry wave number IR intensity selection rules<br># cm**(-1) km/mol IR RAMAN<br>7 a' 112.87 1.03787 YES YES<br>8 a" 151.96 17.49906 YES YES<br>9 a' 221.73 3.57017 YES YES<br>10 a" 235.14 0.94908 YES YES<br>11 a' 256.82 2.93944 YES YES<br>12 a' 562.39 36.37347 YES YES<br>13 a" 617.88 132.10253 YES YES<br>14 a' 618.37 34.22088 YES YES<br>15 a' 1085.44 21.33025 YES YES<br><br>zero point VIBRATIONAL energy : 0.0089030 Hartree
RHF-UCCSD(T)/aug-cc-pVTZ(-PP)	
Cartesian coordinates	5<br>UCCSD(T)/USERDEF ENERGY=-457.86466570<br>Ir -0.2391045032 -0.1211980871 0.0000000000<br>N -0.1850065394 -1.7432729342 0.0000000000<br>F -0.4473897992 0.4561078126 -1.7831466137<br>F -0.4473897992 0.4561078126 1.7831466137<br>F 1.3188906412 0.9522553961 0.0000000000<br><br>T1 diagnostic: 0.02898143<br>D1 diagnostic: 0.10115151
Vibrational data (^{14}N)	Vibration Wavenumber<br>Nr [1/cm]<br>1 104.89<br>2 179.90<br>3 225.03<br>4 240.28<br>5 269.34<br>6 607.25<br>7 650.22<br>8 652.63<br>9 1126.20<br><br>Zero point energy: 0.00923966 [H] 2027.87 [1/CM] 24.26 [KJ/MOL]
Vibrational data (^{15}N)	Vibration Wavenumber<br>Nr [1/cm]<br>1 104.55<br>2 178.91<br>3 223.49<br>4 235.91<br>5 266.43<br>6 607.25

```
   7              650.20
   8              652.60
   9             1090.65

Zero point energy:  0.00913544 [H]     2005.00 [1/CM]       23.99 [KJ/MOL]
```

$FNIrF_2$

$FNIrF_2$ ($^2A'$)

UB3LYP/def2-QZVP

Cartesian coordinates

```
5
Energy = -458.5706094499
F     1.3049440   -0.2086339   -1.7143579
Ir    0.5089844   -0.2782058    0.0000000
F     1.3049440   -0.2086339    1.7143579
N    -1.1997081   -0.2449976    0.0000000
F    -1.9191644    0.9404713    0.0000000
```

Vibrational data (^{14}N)

```
#  mode     symmetry     wave number   IR intensity    selection rul
#                         cm**(-1)        km/mol         IR     RAMA
     7        a'            109.96         8.56509       YES     YES
     8        a"            120.72         0.04914       YES     YES
     9        a'            169.48         7.65746       YES     YES
    10        a'            288.55         1.29362       YES     YES
    11        a"            420.39        48.32981       YES     YES
    12        a'            623.28        45.70357       YES     YES
    13        a'            703.06       215.95348       YES     YES
    14        a"            741.67        21.54040       YES     YES
    15        a'            961.67       124.18477       YES     YES

zero point VIBRATIONAL energy  :      0.0094288  Hartree
```

Vibrational data (^{15}N)

```
#  mode     symmetry     wave number   IR intensity    selection rules
#                         cm**(-1)        km/mol         IR     RAMAN
     7        a'            109.82         8.55889       YES     YES
     8        a"            120.71         0.04946       YES     YES
     9        a'            169.40         7.64199       YES     YES
    10        a'            285.57         1.34575       YES     YES
    11        a"            410.25        45.29440       YES     YES
    12        a'            623.08        44.12064       YES     YES
    13        a'            691.43       205.56301       YES     YES
    14        a"            739.31        23.11765       YES     YES
    15        a'            933.63       122.89715       YES     YES

zero point VIBRATIONAL energy  :      0.0094288  Hartree
```

UBP86/def2-QZVP

Cartesian coordinates

```
5
Energy = -458.9269467684
F     1.3324608   -0.2155969   -1.7032235
Ir    0.5071831   -0.2869029    0.0000000
F     1.3324608   -0.2155969    1.7032235
N    -1.1989080   -0.2344858    0.0000000
F    -1.9731967    0.9525824    0.0000000
```

Vibrational data (^{14}N)

```
#  mode     symmetry     wave number   IR intensity    selection rules
#                         cm**(-1)        km/mol         IR     RAMAN
     7        a'            106.68         6.01777       YES     YES
     8        a"            112.60         0.09537       YES     YES
     9        a'            162.30         4.93230       YES     YES
    10        a'            294.81         0.47866       YES     YES
    11        a"            338.94         8.65496       YES     YES
    12        a'            614.35        27.14428       YES     YES
    13        a'            641.54       272.92351       YES     YES
    14        a"            658.52        68.49828       YES     YES
    15        a'            903.35        83.94606       YES     YES

zero point VIBRATIONAL energy  :      0.0087325  Hartree
```

$FNIrF_2$ ($^2A''$)

UBP86/def2-QZVP

Cartesian coordinates

```
5
Energy = -458.9390808806
F     1.1447808   -0.1935728   -1.7853805
Ir    0.6156118   -0.2355852    0.0000000
F     1.1447808   -0.1935728    1.7853805
N    -1.1214705   -0.3346896    0.0000000
F    -1.7837028    0.9574204    0.0000000
```

Vibrational data (^{14}N)

```
#  mode     symmetry     wave number   IR intensity    selection rules
#                         cm**(-1)        km/mol         IR     RAMAN
     1                       -0.00         0.00000        -       -
     2                       -0.00         0.00000        -       -
     3                       -0.00         0.00000        -       -
     4                        0.00         0.00000        -       -
     5                        0.00         0.00000        -       -
     6                        0.00         0.00000        -       -
     7        a"            106.72         0.37770       YES     YES
     8        a'            154.44         2.49710       YES     YES
     9        a'            168.09         0.94280       YES     YES
    10        a"            304.06         0.36940       YES     YES
    11        a'            327.56         3.58129       YES     YES
    12        a'            632.47        89.63256       YES     YES
    13        a"            666.97       128.03929       YES     YES
    14        a'            679.53       222.48041       YES     YES
    15        a'            867.79        22.71469       YES     YES

zero point VIBRATIONAL energy  :      0.0089023  Hartree
```

Vibrational data (^{15}N)	# mode symmetry wave number IR intensity selection rules<br># cm**(-1) km/mol IR RAMAN<br>7 a" 106.69 0.37653 YES YES<br>8 a' 154.44 2.49700 YES YES<br>9 a' 168.05 0.94304 YES YES<br>10 a" 295.95 0.35557 YES YES<br>11 a' 325.46 3.15544 YES YES<br>12 a' 631.39 111.49350 YES YES<br>13 a" 666.97 128.04743 YES YES<br>14 a' 667.30 192.48109 YES YES<br>15 a' 840.89 19.45455 YES YES<br><br>zero point VIBRATIONAL energy : 0.0087872 Hartree
UB3LYP/def2-QZVP	
Cartesian coordinates	5<br>Energy = -458.5794076515<br>F 1.1400249 -0.1903900 -1.7820358<br>Ir 0.6090333 -0.2350006 0.0000000<br>F 1.1400249 -0.1903900 1.7820358<br>N -1.1286061 -0.3226483 0.0000000<br>F -1.7604770 0.9384289 0.0000000
Vibrational data (^{14}N)	# mode symmetry wave number IR intensity selection rules<br># cm**(-1) km/mol IR RAMAN<br>7 a" 109.31 0.37819 YES YES<br>8 a' 157.91 4.68500 YES YES<br>9 a' 168.71 1.50875 YES YES<br>10 a" 302.70 0.76878 YES YES<br>11 a' 346.28 2.34988 YES YES<br>12 a' 641.66 73.34058 YES YES<br>13 a" 675.11 155.12460 YES YES<br>14 a' 779.10 224.13031 YES YES<br>15 a' 880.82 45.04697 YES YES<br><br>zero point VIBRATIONAL energy : 0.0092530 Hartree
Vibrational data (^{15}N)	# mode symmetry wave number IR intensity selection rules<br># cm**(-1) km/mol IR RAMAN<br>7 a" 109.28 0.37624 YES YES<br>8 a' 157.91 4.68528 YES YES<br>9 a' 168.67 1.50972 YES YES<br>10 a" 294.62 0.74311 YES YES<br>11 a' 343.92 2.07110 YES YES<br>12 a' 641.37 76.11434 YES YES<br>13 a" 675.10 155.14057 YES YES<br>14 a' 765.03 213.65419 YES YES<br>15 a' 852.96 41.60832 YES YES<br><br>zero point VIBRATIONAL energy : 0.0091328 Hartree
ROHF-UCCSD(T)/aug-cc-pVTZ(-PP)	
Single Point<br>B3LYP minimum	5<br>Energy = -457.82700748<br>F 1.1400249 -0.1903900 -1.7820358<br>Ir 0.6090333 -0.2350006 0.0000000<br>F 1.1400249 -0.1903900 1.7820358<br>N -1.1286061 -0.3226483 0.0000000<br>F -1.7604770 0.9384289 0.0000000<br><br>T1 diagnostic: 0.03488436<br>D1 diagnostic: 0.13687633

F_2NIrF

F_2NIrF ($^2A'$)	
UBP86/def2-QZVP	
Cartesian coordinates	5<br>Energy = -458.8629945639<br>F 1.5482979 -0.4917268 -1.0791222<br>N 0.6755083 -0.2168063 0.0000000<br>F 1.5482979 -0.4917268 1.0791222<br>Ir -0.9922915 0.3134368 0.0000000<br>F -2.7798124 0.8868231 0.0000000
Vibrational data (^{14}N)	# mode symmetry wave number IR intensity selection rules<br># cm**(-1) km/mol IR RAMAN<br>7 a' 61.67 1.77356 YES YES<br>8 a" 112.23 2.42212 YES YES<br>9 a" 307.81 0.00019 YES YES<br>10 a' 376.03 12.13799 YES YES<br>11 a" 553.69 177.98760 YES YES<br>12 a' 562.80 1.43913 YES YES<br>13 a' 584.97 35.49821 YES YES<br>14 a' 647.62 134.52596 YES YES<br>15 a' 990.44 413.55337 YES YES<br><br>zero point VIBRATIONAL energy : 0.0095621 Hartree
UB3LYP/def2-QZVP	
Cartesian coordinates	5<br>Energy = -458.5097621591<br>F 1.5344514 -0.4909063 -1.0645917<br>N 0.6949504 -0.2219223 0.0000000<br>F 1.5344514 -0.4909063 1.0645917<br>Ir -0.9873562 0.3162936 0.0000000<br>F -2.7764969 0.8874415 0.0000000

Vibrational data (^{14}N)

```
#  mode     symmetry     wave number   IR intensity    selection rules
#                         cm**(-1)        km/mol         IR      RAMAN
     7        a'            50.96         2.62706       YES     YES
     8        a"           124.94         3.95268       YES     YES
     9        a"           323.40         0.42925       YES     YES
    10        a'           385.43        10.19836       YES     YES
    11        a'           561.60         1.00217       YES     YES
    12        a'           623.95        11.67796       YES     YES
    13        a'           652.35       155.26240       YES     YES
    14        a"           751.42       162.06913       YES     YES
    15        a'          1036.12       376.49589       YES     YES

zero point VIBRATIONAL energy  :      0.0102749  Hartree
```

F_2NIrF ($^4A''$)

UBP86/def2-QZVP

Cartesian coordinates

```
5
Energy = -458.8266783106
F     1.5089255   -0.5623194   -1.1060538
N     0.8217645    0.0091915    0.0000000
F     1.5089255   -0.5623194    1.1060538
Ir   -1.0371870    0.1922373    0.0000000
F    -2.8024284    0.9232099    0.0000000
```

Vibrational data (^{14}N)

```
#  mode     symmetry     wave number   IR intensity    selection rules
#                         cm**(-1)        km/mol         IR      RAMAN
     7        a'            78.74         4.11158       YES     YES
     8        a"            80.02         3.72134       YES     YES
     9        a"           194.24         0.18452       YES     YES
    10        a'           233.53         0.04359       YES     YES
    11        a'           449.06         2.58740       YES     YES
    12        a'           559.11        58.96818       YES     YES
    13        a'           595.52        74.70332       YES     YES
    14        a"           658.07       150.16710       YES     YES
    15        a'           853.08       331.16844       YES     YES
```

UB3LYP/def2-QZVP

Cartesian coordinates

```
5
Energy = -458.4779953696
F     1.4948764   -0.6022118   -1.0835070
N     0.8885042   -0.0334061    0.0000000
F     1.4948764   -0.6022118    1.0835070
Ir   -1.0191132    0.4341641    0.0000000
F    -2.8591438    0.8036657    0.0000000
```

Vibrational data (^{14}N)

```
#  mode     symmetry     wave number   IR intensity    selection rules
#                         cm**(-1)        km/mol         IR      RAMAN
     7        a'            83.93         3.99938       YES     YES
     8        a"           114.99         1.62177       YES     YES
     9        a'           246.08         0.69189       YES     YES
    10        a'           406.78        11.29014       YES     YES
    11        a"           407.88         9.33163       YES     YES
    12        a'           584.56         5.02530       YES     YES
    13        a'           650.39       130.64898       YES     YES
    14        a"          1001.55       292.75634       YES     YES
    15        a'          1084.75       267.05364       YES     YES
```

$NIrF_2$

$NIrF_2$ ($^1A_1$)

UBP86/def2-QZVP

Cartesian coordinates

```
4
Energy = -359.1012494309
Ir    0.0000000    0.0000000   -0.0765255
N     0.0000000    0.0000000   -1.6908691
F    -1.6251573    0.0000000    0.8836950
F     1.6251573    0.0000000    0.8836950
```

Vibrational data (^{14}N)

```
#  mode     symmetry     wave number   IR intensity    selection rules
#                         cm**(-1)        km/mol         IR      RAMAN
     7        a1           143.42         3.05471       YES     YES
     8        b1           211.05         5.54659       YES     YES
     9        b2           236.94         2.33622       YES     YES
    10        b1           628.70       105.81544       YES     YES
    11        a1           629.74        51.28666       YES     YES
    12        a1          1179.79        30.05040       YES     YES

zero point VIBRATIONAL energy  :      0.0069020  Hartree
```

UB3LYP/def2-QZVP

Cartesian coordinates

```
4
Energy = -358.7910333724
Ir    0.0000000    0.0000000   -0.0819104
N     0.0000000    0.0000000   -1.6803279
F    -1.6217495    0.0000000    0.8811169
F     1.6217495    0.0000000    0.8811169
```

Vibrational data (^{14}N)

```
#  mode     symmetry     wave number   IR intensity    selection rules
#                         cm**(-1)        km/mol         IR      RAMAN
     7        a1           143.75         4.98222       YES     YES
     8        b1           216.38         6.47647       YES     YES
     9        b2           240.74         4.53107       YES     YES
    10        a1           636.19        62.85175       YES     YES
    11        b1           638.94       116.16148       YES     YES
    12        a1          1223.85        34.92929       YES     YES

zero point VIBRATIONAL energy  :      0.0070620  Hartree
```

NIrF

NIrF (^{2}A')	
UBP86/def2-QZVP	
Cartesian coordinates	3<br>Energy = -259.1595662934<br>Ir -0.1550380 0.0071461 0.0000000<br>N -1.0118928 -1.3789176 0.0000000<br>F 1.1669308 1.3717715 0.0000000
Vibrational data (^{14}N)	# mode symmetry wave number IR intensity selection rules<br># cm**(-1) km/mol IR RAMAN<br>7 a' 45.92 4.48934 YES YES<br>8 a' 622.32 102.29311 YES YES<br>9 a' 1151.56 45.60841 YES YES<br><br>zero point VIBRATIONAL energy : 0.0041458 Hartree
UB3LYP/def2-QZVP	
Cartesian coordinates	3<br>Energy = -258.8968248209<br>Ir -0.0672616 -0.0673564 0.0000000<br>N -1.0635023 -1.3434876 0.0000000<br>F 1.1307639 1.4108441 0.0000000
Vibrational data (^{14}N)	# mode symmetry wave number IR intensity selection rules<br># cm**(-1) km/mol IR RAMAN<br>7 a' 31.03 6.01178 YES YES<br>8 a' 629.62 124.10892 YES YES<br>9 a' 1184.57 50.37776 YES YES

Supporting Information References

[1] TURBOMOLE GmbH, *TURBOMOLE V7.0.1*, **2015**.

[2] a) A. D. Becke, *Phys. Rev. A* **1988**, *38*, 3098; b) J. P. Perdew, *Phys. Rev. B* **1986**, *33*, 8822.

[3] a) S. H. Vosko, L. Wilk, M. Nusair, *Can. J. Phys.* **1980**, *58*, 1200; b) C. Lee, W. Yang, R. G. Parr, *Phys. Rev. B* **1988**, *37*, 785; c) A. D. Becke, *J. Chem. Phys.* **1993**, *98*, 5648; d) P. J. Stephens, F. J. Devlin, C. F. Chabalowski, M. J. Frisch, *J. Phys. Chem.* **1994**, *98*, 11623.

[4] a) F. Weigend, F. Furche, R. Ahlrichs, *J. Chem. Phys.* **2003**, *119*, 12753; b) F. Weigend, R. Ahlrichs, *Phys. Chem. Chem. Phys.* **2005**, *7*, 3297.

[5] D. Andrae, U. Huermann, M. Dolg, H. Stoll, H. Preu, *Theor. Chim. Acta* **1990**, *77*, 123.

[6] H.-J. Werner, P. J. Knowles, G. Knizia, F. R. Manby, M. Schütz, P. Celani, W. Györffy, D. Kats, T. Korona, R. Lindh, A. Mitrushenkov, G. Rauhut, K. R. Shamasundar, T. B. Adler, R. D. Amos, S. J. Bennie, A. Bernhardsson, A. Berning, D. L. Cooper, M. J. O. Deegan, A. J. Dobbyn, F. Eckert, E. Goll, C. Hampel, A. Hesselmann, G. Hetzer, T. Hrenar, G. Jansen, C. Köppl, S. J. R. Lee, Y. Liu, A. W. Lloyd, Q. Ma, R. A. Mata, A. J. May, S. J. McNicholas, W. Meyer, T. F. Miller III, M. E. Mura, A. Nicklass, D. P. O'Neill, P. Palmieri, D. Peng, K. Pflüger, R. Pitzer, M. Reiher, T. Shiozaki, H. Stoll, A. J. Stone, R. Tarroni, T. Thorsteinsson, M. Wang, M. Welborn, *MOLPRO, version 2019.2, a package of ab initio programs*.

[7] K. A. Peterson, D. Figgen, M. Dolg, H. Stoll, *J. Chem. Phys.* **2007**, *126*, 124101.

[8] D. Figgen, K. A. Peterson, M. Dolg, H. Stoll, *J. Chem. Phys.* **2009**, *130*, 164108.

[9] R. A. Kendall, T. H. Dunning, R. J. Harrison, *J. Chem. Phys.* **1992**, *96*, 6796.

[10] a) T. H. Dunning, *J. Chem. Phys.* **1989**, *90*, 1007; b) W. A. de Jong, R. J. Harrison, D. A. Dixon, *J. Chem. Phys.* **2001**, *114*, 48; c) N. B. Balabanov, K. A. Peterson, *J. Chem. Phys.* **2005**, *123*, 64107.

[11] O. M. Wilkin, N. Harris, J. F. Rooms, E. L. Dixon, A. J. Bridgeman, N. A. Young, *J. Phys. Chem. A* **2018**, *122*, 1994.

[12] O. Ruff, W. Menzel, *Z. Anorg. Allg. Chem.* **1934**, *217*, 85.

[13] W. Maya, *Inorg. Chem.* **1964**, *3*, 1063.

[14] A. Allan, J.L. Duncan, J.H. Holloway, D.C. McKean, *J. Mol. Spectrosc.* **1969**, *31*, 368.
